# Joint imputation and deconvolution of gene expression across spatial transcriptomics platforms

**DOI:** 10.1101/gr.280555.125

**Published:** 2025-12

**Authors:** Hongyu Zheng, Hirak Sarkar, Benjamin J. Raphael

**Affiliations:** 1 Department of Computer Science, Princeton University, Princeton, New Jersey 08540, USA;; 2 Ludwig Cancer Institute, Princeton Branch, Princeton University, Princeton, New Jersey 08540, USA

## Abstract

Spatially resolved transcriptomics (SRT) technologies measure gene expression across thousands of spatial locations within a tissue slice. Multiple SRT technologies are currently available and others are in active development, with each technology having varying spatial resolution (subcellular, single-cell, or multicellular regions), gene coverage (targeted vs. whole-transcriptome), and sequencing depth per location. For example, the widely used 10x Genomics Visium platform measures whole transcriptomes from multiple-cell-sized spots, whereas the 10x Genomics Xenium platform measures a few hundred genes at subcellular resolution. A number of studies apply multiple SRT technologies to slices that originate from the same biological tissue. Integration of data from different SRT technologies can overcome limitations of the individual technologies, enabling the imputation of expression from unmeasured genes in targeted technologies and/or the deconvolution of admixed expression from technologies with lower spatial resolution. Here, we introduce Spatial Integration for Imputation and Deconvolution (SIID), an algorithm to reconstruct a latent spatial gene expression matrix from a pair of observations from different SRT technologies. SIID leverages a spatial alignment and uses a joint nonnegative factorization model to accurately impute missing gene expression and infer gene expression signatures of cell types from admixed SRT data. In simulations involving paired SRT data sets from different technologies (e.g., Xenium and Visium), SIID shows superior performance in reconstructing spot-to-cell-type assignments, recovering cell type–specific gene expression and imputing missing data compared to contemporary tools. When applied to real-world 10x Xenium-Visium pairs from human breast and colon cancer tissues, SIID achieves highest performance in imputing holdout gene expression.

Spatially resolved transcriptomics (SRT) technologies have transformed the study of tissue biology by enabling the simultaneous measurement of gene expression at thousands to hundreds of thousands of locations within a tissue section, along with the spatial coordinates of each location. SRT technologies allow researchers to study complex spatial gene expression patterns and intricate cellular organization, providing a closer look at the tissue microenvironment and spatial context for a given disease (Rao et al. 2021; Palla et al. 2022). There are multiple SRT technologies currently in use with varying spatial resolution and breadth/depth of gene expression (Rozenblatt-Rosen et al. 2020; Moses and Pachter 2022). For example, in situ capture-based SRT such as Slide-seq (Ståhl et al. 2016; Rodriques et al. 2019) and 10x Genomics Visium measure thousands of genes using barcoded beads (of radius 10–50 *µ*m) on a slide. Here, a single bead captures mRNA molecules from multiple spatially nearby cells and thus the gene expression measurement is for a mixture of multiple cells. In contrast, in situ sequencing- (Ke et al. 2013; Wang et al. 2018) and in situ hybridization-based SRT such as 10x Genomics Xenium (Janesick et al. 2023; Oliveira et al. 2025), merFISH (Chen et al. 2015), CosMx (NanoString), and MERSCOPE (Vizgen) measure the expression for a subset of preselected genes at cellular or subcellular resolution. The number of mRNA molecules measured for each gene varies considerably across different platforms.

Given the varying properties of individual SRT platforms, it is advantageous to integrate information from two or more platforms (e.g., combining information from whole-transcriptome platforms with lower spatial resolution with platforms that measure expression of a limited number of genes at high spatial resolution). Such integration could assist in two tasks: (1) predicting the expression of genes that are missing in the high resolution SRT data sets by referencing its expression in the low resolution SRT data set, a process known as imputation; and (2) inferring the mixture proportion of different cell types in a spot from the low resolution SRT data set by using the high resolution SRT data set as a reference, commonly referred to as deconvolution.

Multiple methods have been introduced to impute gene expression across single-cell sequencing technologies including scRNA-seq and single-cell sequencing assay for transposase accessible chromatin (scATAC-seq) (Lopez et al. 2018; Korsunsky et al. 2019; Stuart et al. 2019; Wu et al. 2021; Cohen Kalafut et al. 2023; Cao et al. 2024). These tools commonly integrate multiple single-cell modalities into a shared latent space, allowing them to impute gene expression in cells on one modality by identifying nearby cells from a complementary modality within the latent space. A common way of modeling the latent space is by finding a low-dimensional factorization of scRNA-seq gene expression matrices (Yang and Michailidis 2016; Argelaguet et al. 2018, 2020; Townes et al. 2019; Liu et al. 2020; Qian et al. 2022).

Similarly, in the spatial transcriptomics domain, the SRT gene expression is traditionally imputed by integrating with a reference scRNA-seq data set, often disregarding the spatial information inherent in the SRT data set, treating it as a nonspatial modality (Lopez et al. 2019). Other methods such as Tangram (Biancalani et al. 2021), SpaGE (Abdelaal et al. 2020), and SpaOTsc (Cang and Nie 2020) impute genes in a SRT data set by learning a mapping between each spatial location and the reference single cells. On the other hand, akin to the modeling of single-cell data sets, several recent methods use low-dimensional factorization (Chidester et al. 2023; Townes and Engelhardt 2023) to model SRT data, taking into account the spatial information. However, these methods are only applicable to a single data set and therefore are not suitable for imputation across multiple SRT modalities.

All of the above mentioned tools used for imputation implicitly assume at least one of the modalities is scRNA-seq. As a result, applying them to impute gene expression across two SRT data sets faces two significant limitations. First, the spatial information in the data sets is ignored by the algorithm, and second, the reference data set is assumed to have single-cell resolution. These limitations have been partially addressed by recently developed spatial alignment based tools, SLAT (Xia et al. 2023) and SANTO (Li et al. 2024), which impute missing gene expression in one of the SRT data sets based on a learned spatial alignment. These tools do not handle platform-specific differences and overlook the need of deconvolution in case of low spatial resolution. To address the issue of admixed gene expression in technologies with multicellular spatial resolution, a number of methods have been developed to deconvolve admixed SRT spots (Andersson et al. 2020; Miller et al. 2022; Vahid et al. 2023). However, these tools are not designed for imputation across two SRT data sets, and furthermore, they assume perfectly matched gene sets across the data sets, making them unusable for imputing gene expression in one SRT data from another.

We introduce **S**patial **I**ntegration for **I**mputation and **D**econvolution (SIID), an algorithm that simultaneously imputes missing genes in a targeted single-cell (or subcellular) resolution SRT data set (e.g., 10x Genomics Xenium) and deconvolves a supercellular resolution whole-transcriptome SRT data set (e.g., 10x Genomics Visium). SIID uses nonnegative matrix factorization (NMF) to construct a latent gene expression matrix from the views obtained by the two SRT technologies. We demonstrate the effectiveness of SIID on both simulated and two paired Xenium-Visium data sets by evaluating the imputed gene expression and the inferred cell type mixtures. On paired 10x Genomics Xenium and Visium data sets from a breast tumor and colorectal tumor, we demonstrate that SIID enables fine-grained cell typing and better characterization of tumor microenvironments.

## Results

### Spatial Integration for Imputation and Deconvolution

SIID performs joint imputation and deconvolution of a single-cell spatially resolved transcriptomics data set (e.g., 10x Genomics Xenium or MERFISH) measuring a targeted gene panel, and a lower resolution spatially resolved transcriptomics data set measuring a large number of genes (e.g., 10x Genomics Visium, Slide-seq, or DBiT-seq). Given an alignment of the two SRT slices, SIID performs a spatially regularized nonnegative matrix factorization, with components shared across slices to capture common biological information. For the sake of clarity and to match data sets where we apply SIID below, we denote these data sets as the Xenium and Visium data sets for the remainder of this manuscript.

**Figure 1.**
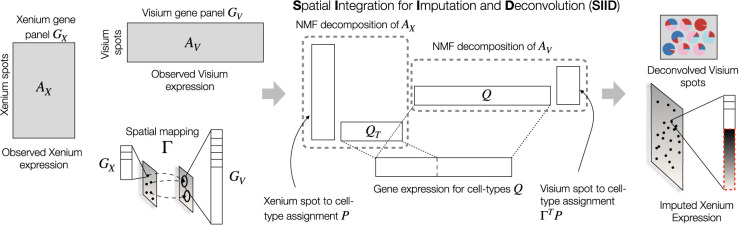
Overview of Spatial Integration for Imputation and Deconvolution (SIID). Given Xenium and Visium expression matrices *A*_*X*_ and *A*_*V*_, respectively, with corresponding gene panels *G*_*X*_ and *G*_*V*_ (with *G*_*X*_ ⊂ *G*_*V*_), and a Xenium to Visium spatial mapping Γ, SIID finds a NMF decomposition of a latent gene count matrix *A*_*U*_, with corresponding factorizations of *A*_*X*_ and *A*_*V*_, into location to latent factor assignment matrix *P* and the gene expression matrix *Q* for latent factors. The estimated parameters are used to impute the absent gene expression in Xenium data and predict the cell type mixture proportions for each Visium spot.

Given the observed Xenium expression matrix *A*_*X*_ with gene panel *G*_*X*_ and the observed Visium expression matrix *A*_*V*_ with gene panel *G*_*V*_ (*G*_*X*_ ⊂ *G*_*V*_), along with the spatial mapping Γ from Xenium spots to Visium spots, SIID constructs *Q*, a shared gene expression matrix for latent factors with a gene panel of *G*_*V*_, and matrix *P* containing the Xenium spot-to-cell-type assignments. The Xenium data are modeled as *A*_*X*_ ≈ *PQ*_*T*_, where *Q*_*T*_ is the subset of *Q* for genes in *G*_*X*_. Similarly, the Visium data are modeled as *A*_*V*_ ≈ (Γ^*T*^*P*)*Q* (Fig. 1). This yields a shared nonnegative matrix factorization of the observations with latent dimension equal to the number of cell types. By constraining the number of latent factors (rows of *Q* or columns of *P*) reflecting underlying cell types, SIID jointly estimates *P* and *Q*, enabling imputation of the absent Xenium genes and deconvolution of the Visium spots.

Compared with prior NMF-based methods, SIID differs in two key aspects. First, most existing methods either work with a single modality or fit two NMFs sharing one of the components. In our model, the NMFs of two SRT data sets share both components via the spatial mapping. Second, by sharing both NMF components, our model learns the cell type assignments for admixed spots in a way that respects spatial information.

### Setup and evaluation

For simulation and evaluation, we use two publicly available data sets of paired Xenium and Visium SRT from adjacent tissue sections: one from a breast cancer (BRCA) pair (Janesick et al. 2023) and another from a colorectal cancer (CRC) pair (Oliveira et al. 2025). Xenium, Visium, and scRNA-seq data for both data sets (and cell type annotation for BRCA) are downloaded and aligned as described in Supplemental Methods C.1. With aligned coordinates, Γ is generated by matching each Xenium spot to its closest Visium spot up to a distance of 100 *µ*m. Furthermore, Supplemental Methods C.2 and Supplemental Table S1 contain detailed data set statistics.

### Imputation setup and holdout evaluation

For both the BRCA and CRC data sets, we divide Xenium panel genes into 10 equal-sized folds. For each fold, we remove the expression of genes in fold from the Xenium data set, then run the imputation algorithm to obtain estimates of these holdout genes. To evaluate the imputation results, for each gene in the fold, we compute the *R*^2^ score between estimated expression for each Xenium cell and the observed counts (which were held out during training). The *R*^2^ score between two vectors (*x*, *y*), also called the coefficient of determination, equals $\mathrm{co}v^{2}(x,y)/\sigma_{x}^{2}\sigma_{y}^{2}$, where cov is the covariance and *σ*^2^ is the variance. The *R*^2^ score for a given model is the average gene-wise holdout *R*^2^ across all 10 folds, where each gene is a holdout exactly once across 10 folds.

### SIID recovers the cell type expression in simulated data set

We evaluate the performance of SIID on a simulated paired Xenium and Visium data set generated from the BRCA data set (Janesick et al. 2023). The evaluation focuses on three aspects: imputation of genes not present in the Xenium data; recovery of the spatial location to cell type assignments; and accuracy of cell type gene expression profiles.

**Figure 2.**
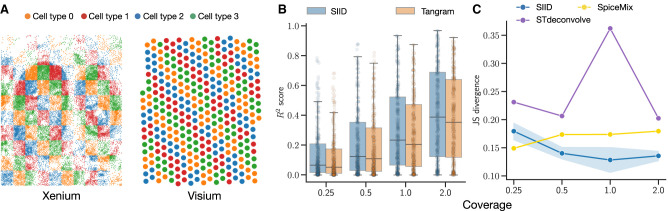
Evaluating SIID on simulated data. (*A*) A simulated Xenium and corresponding Visium data set with gene expression of cell types obtained from a matching scRNA-seq data set (Janesick et al. 2023). (*B*) *R*^2^ of imputed gene expression for holdout genes from SIID and Tangram stratified by coverage. (*C*) Average Jensen-Shannon (JS) divergence between ground truth and predicted cell type mixture proportions predicted by SIID, STdeconvolve, and SpiceMix for simulated Visium data over all spots stratified by coverage.

To simulate a realistic expression profile, we use the gene expression for the scRNA-seq data set and the spatial coordinates from the Xenium and Visium data. For this simulation, we used clusters (see Supplemental Methods B.1) obtained from unsupervised clustering of the scRNA-seq data and define them as cell types. Each Xenium spot is assigned to a cell type using a checkerboard spatial pattern (as described in Fig. 2A; Jackson et al. 2024), where each grid on the checkerboard has a different cell type from its neighbors. We use the average gene expression for individual cell type from the scRNA-seq data set to simulate the gene counts for Xenium spots according to their cell type. Expression data for Visium spots are generated by applying the spatial mapping from Xenium to Visium data (see detailed steps in Supplemental Methods B.1).

We created a variety of simulation data sets by varying (i) the number of grids, *l* on each side of the checkerboard with *l* ∈ {10, 20}, (ii) gene counts generated for each Xenium spots, and (iii) the number of cell types *h*, where *h* ∈ {4, 8, 16}. To simulate Xenium gene expression, we began with the actual vector of UMI counts *N*_*X*_ from the BRCA Xenium data set. This was then scaled using a coverage fraction *ρ* (which we refer to as *coverage*) to create a UMI count vector $N_{X}^{\prime }=\rho N_{X}$, with *ρ* ∈ {0.25, 0.5, 1, 2}, which is used for simulating gene expression for Xenium spots. By varying *l*, *h*, *ρ*, we created in total 24 distinct configurations, each representing varying levels of difficulty. We profiled and compared the run time and memory usage of SIID and Tangram as described in Supplemental Methods B.4 and Supplemental Figure S7. We also studied the effect of additional factors in simulation, namely, spatially variable coverage, noise in spatial mapping matrix, and using platform scaling factor in the imputation results (Supplemental Methods B.3, B.5, B.6; Supplemental Figs. S2–S4, S11).

We evaluated the accuracy of SIID for gene expression imputation and cell type deconvolution in simulations, comparing its performance to Tangram, STdeconvolve, and SpiceMix. For the configuration with *l* = 20 grids and *h* = 8 cell types, SIID achieves superior *R*^2^ scores when compared to Tangram (Biancalani et al. 2021) across different coverage levels (Fig. 2B). On the same simulation configuration, SIID outperforms STdeconvolve and SpiceMix by achieving the lowest average Jensen-Shanon (JS) divergence (Fig. 2C) between the predicted cell type mixture proportion (see Supplemental Methods B.2) and the ground truth. On other simulation configurations (Supplemental Fig. S1), SIID consistently receives the best *R*^2^ scores when compared to Tangram and comparable JS divergence when compared to STdeconvolve, and SpiceMix (see Supplemental Methods B.2). Additionally, we created a challenging simulation scenario by setting grid size *l* = 100 (Supplemental Fig. S16A), where we observed that SIID achieves significant improvement in *R*^2^ score when compared to Tangram (Supplemental Fig. S16B).

### Imputing missing genes in cancer SRT data

To perform benchmarking on BRCA and CRC data sets (Results, “Imputation setup and holdout evaluation”), we ran SIID with number of latent factors *h* = 20 for BRCA and 40 for CRC data sets, entropy regularization with *λ* = 1000, platform scaling enabled, 5000 training epochs and three restarts. For training, we used genes that are available in both Xenium and Visium data sets, excluding the ones in the holdout set. We document the details of hyperparameters in Supplemental Methods C.3.

We benchmarked four existing methods (Li et al. 2022) for evaluating the imputation results: Tangram (Biancalani et al. 2021) (in cell and cluster modes), gimVI (Lopez et al. 2019), SLAT (Xia et al. 2023), and SANTO (Li et al. 2024) (with precomputed mapping Γ). However, we do not present results for gimVI (Lopez et al. 2019) due to run time errors. In addition, we present four baseline approaches by using the spatial mapping Γ: Baseline A imputes gene expression for each Xenium cell by simply using the gene expression from the corresponding Visium spot based on the spatial mapping; Baseline B is a variant of Baseline A that takes total count per Xenium cell into account; and Baselines C and D employ *k*-nearest-neighbor based smoothing based on Baselines *A* and *B*, respectively. Detailed procedures for benchmarking are in Supplemental Methods C.4, and run times for benchmarking are in Supplemental Methods C.6 and Supplemental Table S2. In addition, we show that SIID is scalable and runs efficiently with a larger number of latent factors, genes, Xenium spots, or Visium spots (Supplemental Methods C.6; Supplemental Fig. S5).

#### SIID accurately imputes genes in the holdout experiments

Compared to other methods, SIID achieves the best imputation performance on both BRCA and CRC data sets, as measured by average holdout *R*^2^ scores across all 10-folds (Table 1). Among the competing methods, Tangram performs reasonably well when run in cell mode (referred to as Tangram [cell] in Table 1). In addition to *R*^2^ scores, SIID also consistently outperforms other benchmarked methods on four other correlation metrics (Supplemental Methods D.2; Supplemental Table S5; Li et al. 2022). Additionally, SIID shows robust performance in the presence of noise in the spatial mapping matrix (Supplemental Methods C.5; Supplemental Fig. S6).

**Table 1.** Comparison of average holdout *R*^2^ scores across methods

		Tangram				Baselines			
Data set	SIID	Cell mode	Cluster mode	SLAT	SANTO	A	B	C	D
BRCA	**0.2527**	0.1874	0.1556	0.0688	0.1042	0.0373	0.0807	0.0766	0.0664
CRC	**0.2248**	0.1789	0.1382	0.0608	0.0727	0.0325	0.0573	0.0443	0.0412

Bold indicates the best performer.

**Figure 3.**
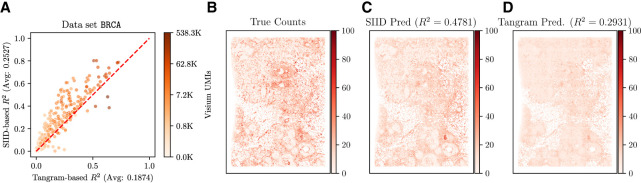
Analysis of SIID imputation on breast cancer (BRCA) data. (*A*) Comparison of holdout *R*^2^ score of SIID (*y*-axis, correlation score averaged over five runs) and Tangram in cell mode (*x*-axis) for each gene in the Xenium panel for the BRCA data set. Each point on the plot corresponds to a single gene, whose color corresponds to the number of Visium UMIs mapped to the gene on a log-scale. Genes *above* the red *y* = *x* line have higher imputation performance for SIID compared to Tangram. (*B*) Expression of gene *ZEB2* in Xenium (ground truth for evaluating imputation), (*C*) SIID prediction of *ZEB2* expression when the gene is held out (*R*^2^ = 0.4781 against the ground truth). (*D*) Tangram prediction of *ZEB2* expression (*R*^2^ = 0.2931 against ground truth). Total counts of each gene over all cells are normalized to 1,000,000, and normalized counts are shown on the same color scale in *B*, *C*, and *D*.

SIID also achieves a higher *R*^2^ score for most of the individual genes compared to the closest competitor (Fig. 3A). For this evaluation, we compared SIID with Tangram (in cell mode) by evaluating the *R*^2^ scores of both methods for each gene in the Xenium panel from the BRCA data set (see Supplemental Fig. S8A for CRC) along with the total UMI counts. We observed that SIID outperforms Tangram on most of the genes, whereas Tangram performs better for a small number of genes. We further visualized the difference of *R*^2^ scores and its relationship to expression level in Supplemental Figure S9 and concluded that there is no clear correlation between them. As SIID is a generative model, we also validated the model by showing its ability to predict the sparsity of holdout genes (Supplemental Fig. S10).

#### SIID recovers spatial pattern of Xenium gene expression

SIID is superior to Tangram in recovering spatial patterns for certain marker genes (Fig. 3B–D; Supplemental Fig. S12; Janesick et al. 2023). In particular, we found that the SIID-imputed expression for *ZEB2*, a well-known oncogenic driver implicated in epithelial-mesenchymal transition (EMT) in breast cancer (di Gennaro et al. 2018; De Coninck et al. 2019), closely mirrors the true tissue structure (*R*^2^ = 0.48) (Fig. 3C), whereas Tangram's imputed expression is more uniformly distributed across the slice (*R*^2^ = 0.29) (Fig. 3D). We present similar results for other marker genes in Supplemental Figure S12.

#### Comparison to imputing with paired scRNA-seq

On imputing Xenium genes, SIID with Visium data outperforms Tangram paired with a scRNA-seq reference (Table 2). SLAT and SANTO are only applicable to SRT data sets and therefore are not included in this comparison. gimVI failed to finish on several runs (details in Supplemental Methods C.4) for both modes and both data sets (Supplemental Table S6). SIID is not specifically designed to impute Xenium gene expression with a nonspatial reference, but Tangram can be run in both cell and cluster mode for this setup. In order to run Tangram on the CRC data set in a reasonable time, we downsampled the data set as described in Supplemental Methods C.4. To emphasize, despite not using the high resolution scRNA-seq data, SIID outperforms Tangram with access to scRNA-seq data, although with a smaller lead.

**Table 2.** Comparison of average holdout *R*^2^ scores using scRNA-Xenium pairing

Data set	SIID (Visium)	Tangram (scRNA, cell)	Tangram (scRNA, cluster)
BRCA	**0.2527**	0.2195	0.2203
CRC	**0.2248**	0.196	0.1822

### Deconvolving cell types in cancer SRT data

We also evaluated SIID's performance in deconvolving the admixed gene expression in Visium data using the paired Xenium data. For deconvolving Visium spots without annotation, we ran the same setup as described in Results, “Imputing missing genes in cancer SRT data” and in Supplemental Methods C.3, with no holdout genes and *λ* = 500 for entropy regularization. Here, we evaluated only with the BRCA data set, as this data set includes annotated cell types for both the Xenium and scRNA-seq data provided by 10x Genomics.

#### SIID recovers most annotated major cell types

**Figure 4.**
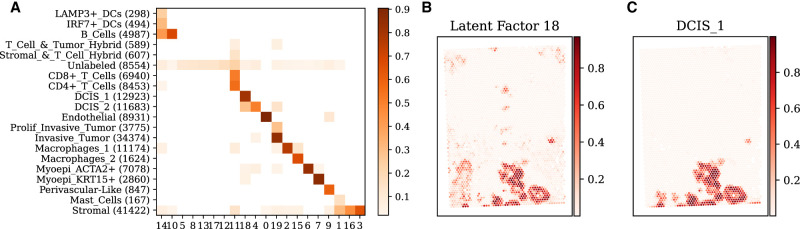
Analysis of SIID deconvolution on breast cancer (BRCA) data. (*A*) Comparison of the clustering of Xenium cells obtained by SIID on the BRCA data set with the cell type annotation provided by 10x Genomics. Each row corresponds to an annotated cell type, with the number of Xenium cells belonging to that type in parentheses. Each column corresponds to a latent factor derived by SIID. Intensity of each grid point is the cosine similarity between the assignments, with similarity scores below 0.02 excluded for visual clarity. (*B*) Spatial distribution of a latent factor inferred by SIID. (*C*) Spatial distribution of deconvolved DCIS_1 cell type from RCTD.

SIID recovers most annotated cell types with more than 1000 cells by mapping them to one or a few latent factors (Fig. 4A). To benchmark this, we trained the model on the BRCA data set. Because the reference annotation assigns each cell to exactly one type, we similarly assigned each Xenium cell to one of 20 latent factors by finding which latent factor contributes the most to its inferred expression; that is, Xenium cell *i* is assigned to latent factor $\arg\max_{j}\;P[i,j]$. We calculated the cosine similarity (Fig. 4A) of this clustering of cells to the cell type annotation provided by 10x Genomics, which contain 19 distinct cell types (besides “Unlabeled“) and some annotations are more granular than others. In addition, we found that the clustering of Xenium cells by SIID is more similar to the 10x Genomics annotation (adjusted Rand index [ARI] = 0.460) than the Leiden clustering (with the same number of clusters) is to the 10x annotations (ARI = 0.347).

#### SIID recovers spatial distribution of deconvolved cell types

SIID's unsupervised deconvolution of Visium spots are visually similar to the supervised deconvolution performed by RCTD (Cable et al. 2022), a leading method for deconvolving admixed SRT data (Fig. 4B,C). For SIID, we trained the model on the BRCA data set (with Xenium and Visium data) and obtained the unsupervised latent factor deconvolution for each Visium spot by computing *M*^*T*^*P* and normalizing by each spot. We ran RCTD (Cable et al. 2022) with Visium and scRNA-seq data annotated with cell types to obtain a supervised cell type deconvolution of Visium spots (cosine similarity plot in Supplemental Fig. S8B). We observed that the spatial distribution of deconvolved DCIS_1 cell type from RCTD (Fig. 4C) and latent factor 18 from SIID (Fig. 4B) have strong spatial agreement. We emphasize that other imputation tools such as Tangram are not capable of determining cell types for spatial location.

### SIID imputation leads to stromal cell subtype discovery in breast cancer Xenium data

**Figure 5.**
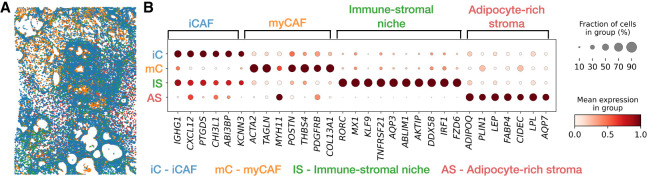
Stromal cell subtype analysis. (*A*) Spatial distribution of stromal cells in Xenium data with four annotated cell subtypes. (*B*) Marker gene expression for the annotated cell subtypes within the stromal cell type in Xenium data.

We used SIID to impute gene expression in the breast cancer Xenium data set and used the imputed expression data to decipher cell subtypes within the subpopulation of 41,422 cells (out of 167,780 total cells in the data set) that were annotated as stromal cells by Janesick et al. (2023). The imputed gene expression from SIID improves clustering (Supplemental Methods C.7; Supplemental Fig. S13) and led to identification of four stromal cell subtypes which we labeled as inflammatory cancer associated fibroblasts (iCAF), myofibroblast-like CAF (myCAF), immune-stromal niche, and adipocyte-rich stroma (Fig. 5A). These annotations were inferred from chosen gene signatures that were previously associated with the corresponding cell type. Specifically, we used curated gene signatures (Wolf et al. 2018) to score each Xenium spot and assigned a label to the cluster that showed the strongest agreement with the signature (Fig. 5B; Supplemental Fig. S15).

The first cluster exhibits upregulation of *CXCL12, PTGDS, KCNN3*, suggesting *CXCL12*-high fibroblasts in the immune infiltrated stroma within the tumor microenvironment (TME). CAF-secreted *CXCL12* (Ahirwar et al. 2018) has been shown to be a major driver within the TME that interacts with the endothelial cells and promotes vascularization. *PTGDS* (prostaglandin D2 synthase) (Jiang et al. 2020; Forsthuber et al. 2024) and *KCNN3*, encoding a potassium channel that regulates the interactions between the immune system with the CAFs (Mohr et al. 2019), are consistent with these immune-interactive stromal cells. Consequently, we refer to this cluster as inflammatory CAFs (iCAFs). The second cluster exhibits upregulation of *ACTA2* (alpha smooth muscle actin), which is an indication of CAFs (Hu et al. 2022). More specifically, the presence of *TAGLN* (transgelin), and *COL13A1* (collagens) points to matrix-remodeling of CAF subtype (Santi et al. 2018), and we therefore annotated this cluster as myfibroblast-like CAF or myCAF. The third cluster shows a *RORC*-driven (Cinnamon et al. 2024), interferon-inducible program with upregulated *MX1, KLF9* (Zhang et al. 2020), and *AQP3* (Charlestin et al. 2022), suggesting an active Th17 (a subset of helper T cells) effector program. The coexistence of *CXCL12*-high CAFs and immune-rich stroma indicates these spots represent a mixture rather than pure stromal cell types. Due to this hybrid nature, we refer to this cluster as an immune–stromal niche. Finally, spots in the fourth cluster coexpress adipocyte markers (*ADIPOQ, PLIN1, CIDEC*) reflecting adipocyte-rich stroma that lead to microvasculature in breast cancer (Zhu et al. 2022). Thus, we refer to this cluster as adipocyte-rich stroma. Spatial colocalization (Fig. 5A) of these cell subtypes shows a clear pattern of stromal subtypes where myCAF cells localize in the interior of iCAFs that constitute the majority of the tissue. Further, we analyzed spatial colocalization of these stromal subtypes with the stromal and T cell hybrid cluster (Supplemental Fig. S13) and observed consistent spatial co-occurrence (Supplemental Methods C.7; Supplemental Fig. S14).

## Discussion

SRT technologies continue to evolve, with new techniques to measure gene expression in physical space with varying spatial resolution, gene panel size, sequencing depth, processing time, and cost. Selecting the most appropriate SRT protocol for profiling a target sample can be challenging. One alternative is to use multiple SRT methods to profile adjacent or nearby slices from the same tissue and combine gene expression information across slices. This approach has the potential to mitigate the limitations of individual modalities. However, most existing methods for integrating data across SRT slices either focus on integration without spatial information—essentially treating SRT data sets as scRNA-seq data sets—or are designed for integrating SRT slices from the same modality and largely ignore potential differences between modalities.

SIID is a new approach of integrating SRT data sets that uses spatial information during integration and simultaneously performs imputation on a high spatial resolution targeted SRT data set and deconvolution on a low spatial resolution SRT data set. We demonstrate SIID on 10x Genomics Xenium and Visium data sets where we infer a latent single-cell whole transcriptome SRT data set that simultaneously imputes the Xenium data to whole transcriptome, recovers gene expression of the cell types, and deconvolves the Visium spots into cell type proportions. We evaluate SIID on both simulated and paired Xenium-Visium data sets where it shows strong performance in both imputation and deconvolution.

SIID will be useful in tissue atlas projects that aim to produce high-resolution multimodal reconstructions of tissues. Due to current costs of SRT platforms, multimodal tissue atlases are mostly large consortium projects. For example, the Human Tumor Atlas Network (HTAN) (de Bruijn et al. 2025) has been profiling tumors with multiple SRT technologies to evaluate tumor heterogeneity in 3D. Multiple SRT technologies are being used in this project; however, no single SRT technology provides both whole-transcriptome coverage and high spatial resolution. SIID enables the integration of two SRT data sets from adjacent tissue slices without using a nonspatial modality such as single-cell RNA sequencing. Thus, both deconvolution and imputation are performed on biologically similar transcriptomes, rather than relying on a transcriptome that may have been altered during single-cell RNA-seq preparation steps (Piwecka et al. 2023). SIID enables downstream analyses such as identifying differentially expressed genes across tissue slices measured with different SRT technologies. As costs of SRT technologies decrease, smaller projects may benefit from SIID's integration of complementary SRT data (such as 10x Visium and 10x Xenium) from the same tissue.

There are several limitations of SIID which are also directions for further improvement. First, SIID models platform-specific differences in expression solely through the platform scaling factors. Thus, our current analyses did not use out-of-Xenium-panel genes that are present in Visium data sets, effectively discarding a substantial fraction of Visium counts (Supplemental Methods C.2). Extending the loss function in SIID to account for the expression differences would enable better utilization of out-of-panel gene expression during inference. Second, our current method is reliant on an accurate spatial mapping matrix Γ, which is not always available. We further discuss the alignment procedure and potential sources of misalignment in Supplemental Methods C.1 and effects of misalignment in both simulation (Supplemental Methods B.3) and BRCA/CRC (Supplemental Methods C.5) data sets. Jointly inferring the spatial alignment Γ by extending existing SRT alignment algorithms (Zeira et al. 2022; Clifton et al. 2023; Liu et al. 2023) might yield better performance. Third, our method currently uses a Poisson count model but can easily be extended to other probability distributions. For example, a very common practice in modeling single-cell RNA-seq data is to assume overdispersion of observed counts. Towards this end, in Supplemental Methods C.8 we describe an extension of SIID that models Visium counts using negative binomial distribution with gene-specific overdispersion. An alternative approach is to model dropouts with zero-inflated distributions (Ridout et al. 2001; Yau et al. 2003); however, careful implementation is required to avoid overfitting the data given the large number of parameters. Fourth, SIID contains a number of hyperparameters whose values need to be selected. We selected the number of hidden dimensions *h* through exploratory data analysis with unsupervised clustering but found that SIID is robust with larger values of *h* (Supplemental Methods C.3, D.1). Analysis of hyperparameter values showed small variation in performance for varying regularization parameter *λ* and number of epochs. Multiple restarts provide a boost to performance and are always recommended (Supplemental Tables S3, S4). In future extensions, we will explore venues of automatically inferring these hyperparameters depending on the use case. Finally, SIID has thus far been tested only on 10x Genomics Xenium and Visium paired data due to the lack of publicly available paired data from other spatial platforms. Whereas the underlying algorithm of SIID is applicable to other imaging and sequencing-based spatial platforms, the performance of SIID on other platforms is not yet known.

SIID is a robust and interpretable method that is scalable and flexible to handle new SRT technologies, such as the recent Xenium 5K and Visium HD (Oliveira et al. 2025) that measure a large panel of genes at subcellular resolution. These and other high resolution data sets are typically more sparse than low resolution or small-panel data sets. Integration methods such as SIID can be adapted to improve coverage of these sparse data sets, allowing for accurate characterization of the transcriptome of the target tissue.

## Methods

### Representing paired spatially resolved transcriptomics data

We represent a spatially resolved transcriptomics data set by a pair (*A*, *S*), where *A* ∈ ℕ^|*S*|×|*G*|^ is a gene count matrix and *S* ∈ ℝ^|*S*|×2^ is the two-dimensional physical coordinates for each spatial location and where *G* represents the set of genes measured in the SRT experiment. Suppose we are given two SRT tissue slices (*A*_*X*_, *S*_*X*_) and (*A*_*V*_, *S*_*V*_) from the same tissue measured with two different protocols which we denote by *X* and *V*. We assume that *X* has higher spatial resolution and a limited set of genes (small-panel), whereas *V* has lower spatial resolution but contains a superset of measured genes (large-panel), meaning |*S*_*X*_| ≫ |*S*_*V*_| and *G*_*X*_ ⊂ *G*_*V*_. In this manuscript, *X* and *V* originate from 10x Xenium and 10x Visium platforms, respectively.

We further assume that there exists an alignment between (*A*_*X*_, *S*_*X*_) and (*A*_*V*_, *S*_*V*_); that is, there is a spot-spot correspondence between two slices as computed from any method that aligns two SRT data sets (Zeira et al. 2022; Clifton et al. 2023; Jones et al. 2023; Liu et al. 2023; Li et al. 2024; Tang et al. 2024). Given the spot-spot correspondence, the coordinates *S*_*X*_ and *S*_*V*_ can be transformed to represent locations in a shared coordinate system between the SRT data sets. After such spatial transformation, the spot-to-spot correspondence matrix is represented with a binary mapping matrix $\Gamma \in {\left\{0,1\right\}}^{\left|S_{X}|\times |S_{V}\right|}$, where Γ[*i*, *j*] = 1 if and only if the Xenium spot *i* is mapped to the Visium spot *j*. Our proposed framework works with any arbitrary Γ.

### A shared cell type model between paired SRTs

We assume that *A*_*X*_ and *A*_*V*_ are sampled from a latent gene count matrix *A*_*U*_ based on the following assumptions:

- There exists a latent SRT data set *U* represented with (*A*_*U*_, *S*_*X*_), where $A_{U}\in N^{\left|S_{X}|\times |G_{V}\right|}$ is the latent gene expression matrix. By construction, *U* has the same spatial coordinates as *X* and the same gene set as *V*.
- *A*_*U*_ follows a Poisson distribution with mean $\bar{A_{U}}$: $A_{U}∼\mathrm{Pois}(\bar{A_{U}})$. As *A*_*X*_ is a submatrix of *A*_*U*_, it naturally follows that $A_{X}∼\mathrm{Pois}(\bar{A_{X}})$, where $\bar{A_{X}}$ is a submatrix of $\bar{A_{U}}$ with columns restricted to *G*_*X*_.
- *A*_*V*_ follows a Poisson distribution with mean $\bar{A_{V}}=K^{T}\bar{A_{U}}$, where $K\in R_{\geq 0}^{\left|S_{X}|\times |S_{V}\right|}$ and *K* ○ (1 − Γ) = 0, where ○ represents the Hadamard product. *K*[*i*, *j*] represents the weight of contribution of a Xenium spot *i* to construct the Visium spot *j* and is zero wherever Γ[*i*, *j*] = 0.
- $\bar{A_{U}}$ has a low-dimensional nonnegative matrix factorization, which we denote as $\bar{A_{U}}=WH$, with $W\in R_{\geq 0}^{|S_{X}|\times h}$ and $H\in R_{\geq 0}^{h\times |G_{V}|}$, where *h* is the number of latent factors. This implies *A*_*X*_ has Poisson mean $\bar{A_{X}}=WH_{X}$, where *H*_*X*_ is a submatrix of *H* with columns restricted to *G*_*X*_, and *A*_*V*_ has Poisson mean $\bar{A_{V}}=K^{T}WH$.

With these assumptions, we formally state our inference problem as follows.

#### Paired NMF Inference Problem

Given a pair of SRT slices $(A_{X}\in N^{\left|S_{X}|\times |G_{X}\right|},S_{X}\in R^{|S_{X}|\times 2}),$ $(A_{V}\in$ $N^{\left|S_{V}|\times |G_{V}\right|}$, $S_{V}\in R^{|S_{V}|\times 2})$, and spatial mapping matrix $\Gamma \in {\left\{0,1\right\}}^{\left|S_{X}|\times |S_{V}\right|}$, find nonnegative matrices $W\in R_{\geq 0}^{|S_{X}|\times h}$ $H\in R_{\geq 0}^{h\times |G_{V}|}$, and $K\in R_{\geq 0}^{\left|S_{X}|\times |S_{V}\right|}$ that solve the following problem:

(1) $$\begin{array}{c} \underset{W,H,K}{\min}\;\mathrm{PoiLoss}(A_{X};WH_{X})+\mathrm{PoiLoss}(A_{V};K^{T}WH)\\ \mathrm{subject}\;\mathrm{to}\;W,H,K\geq 0\;\mathrm{and}\;K\circ (1-\Gamma )=0. \end{array}$$

Here, *K* corresponds to mixture weights matrix, where *K*[*i*, *j*] represents the contribution of Xenium spot *i* to the Visium spot *j* and PoiLoss(Y;Z)=∑(Z−YlogZ) is the negative log-likelihood for observing Y∼Pois(Z).

#### Count-scaled reparameterization

For numerical stability and better interpretation, we solve a reparameterized version of the Paired NMF Inference Problem. Recall $\bar{A_{U}}$ has a low-dimensional NMF $\bar{A_{U}}=WH$, from which we derive estimated expression $\bar{A_{X}}$ and $\bar{A_{V}}$. We rewrite this factorization with an alternative formulation where $\bar{A_{U}}=\mathrm{diag}(N)PQ$ and where

- $N\in R_{\geq 0}^{\left|S_{X}\right|}$ is a vector where *N*[*i*] is the inferred total gene counts for spot *i* in *U*, and $\mathrm{diag}(N)\in R_{\geq 0}^{\left|S_{X}|\times |S_{X}\right|}$ is a matrix whose diagonal elements are *N*,
- $P\in R_{\geq 0}^{|S_{X}|\times h}$, where each row *P*[*i*] is the normalized latent factor composition of spot *i* in *U*, that is, $P1_{h}=1_{\left|S_{X}\right|}$,
- $Q\in R_{\geq 0}^{h\times |G_{V}|}$, where each row *Q*[*j*] is the normalized expression of latent factor *j* in *G*_*V*_, that is, $Q1_{\left|G_{V}\right|}=1_{h}$.

We first show that the reparameterized problem is equivalent to the original.

Lemma 1. Given $\bar{A_{U}}=WH$, there exists *P*, *Q*, *N* as defined above such that $\bar{A_{U}}=\mathrm{diag}(N)PQ$, and vice versa.

Proof (see Supplemental Methods A).

From $\bar{A_{U}}=\mathrm{diag}(N)PQ$, we have $\bar{A_{X}}=\mathrm{diag}(N)PQ_{X}$, where *Q*_*X*_ is a submatrix of *Q* with columns restricted to *G*_*X*_ . We next reparameterize *K*, the mixture weight. We define *M* = diag(*N*)*K*, and thus, $\bar{A_{V}}=K^{T}\mathrm{diag}(N)PQ=M^{T}PQ$. *M* replaces *K* as the variable for inference and has identical constraints of *K*: *M* ≥ 0, *M* ○ (1 − Γ) = 0. The reparameterized version is formally stated as follows.

#### Reparameterized Paired NMF Inference

Given a pair of SRT slices $\left(A_{X}\in N^{\left|S_{X}|\times |G_{X}\right|},S_{X}\in R^{|S_{X}|\times 2}\right)$, $\left(A_{V}\in N^{\left|S_{V}|\times |G_{V}\right|},S_{V}\in R^{|S_{V}|\times 2}\right)$, and spatial mapping matrix $\Gamma \in {\left\{0,1\right\}}^{\left|S_{X}|\times |S_{V}\right|}$, find nonnegative matrices $P\in R_{\geq 0}^{|S_{X}|\times h},Q\in R_{\geq 0}^{h\times |G_{V}|},N\in R_{\geq 0}^{\left|S_{X}\right|}$ and $M\in R_{\geq 0}^{\left|S_{X}|\times |S_{V}\right|}$ that solve the following problem:

(2) $$\begin{array}{rl} & \underset{P,Q,N,M}{\min}\;\mathrm{PoiLoss}(A_{X};\;\mathrm{diag}(N)PQ_{X})+\mathrm{PoiLoss}(A_{V};M^{T}PQ)\\ & \mathrm{subject}\;\mathrm{to}\;P,Q,N,M\geq 0,P1_{h}=1_{\left|S_{X}\right|},Q1_{\left|G_{V}\right|}=1_{h},M\circ (1-\Gamma )=0 \end{array}$$

*M* corresponds to the mixture weights, with *M*[*i*, *j*] being the contribution of gene counts from Xenium spot *i* to Visium spot *j*, and, as above, PoiLoss(Y;Z)=∑(Z−YlogZ) is the negative log-likelihood for observing Y∼Pois(Z).

The reparameterized problem allows easier interpretation of the inferred model.

### Implementation, parameter inference, and imputing missing genes

We implement SIID to solve the Reparameterized Paired NMF Inference problem (Eq. 2). SIID solves the optimization problem in PyTorch (https://pytorch.org/) using gradient descent to optimize the model parameters. We use Adam optimizer and train for 5000 epochs, with a learning rate of 0.05.

#### Parameters of the model and ℓ_2_ regularization

Because *P* and *Q* are row-normalized, we represent them as softmaxed matrices. Similarly, *N*, *M* are represented as exponentiated matrices. For numerical stability reasons, we place a ℓ_2_ regularization with weight 10^−5^ on the parameters of the model (*P*, *Q* before softmax, *N*, *M* before exponentiation) (Biancalani et al. 2021).

#### Platform scaling

Our model assumes existence of a latent gene expression matrix *A*_*U*_ that, in turn, generates observations *A*_*X*_ and *A*_*V*_. In practice, if the two SRT data sets are generated from different SRT platforms, we expect platform-specific effects that should be modeled on top of the latent gene expression. Observing that the same gene could be expressed at different rates in different SRT data sets (Cable et al. 2022), we introduce a gene-wise scaling $\phi \in R_{>0}^{\left|G_{V}\right|}$, as a multiplier to the columns (corresponding to genes) of $\bar{A_{V}}$ (to reduce nonidentifiability in practice, we fix *ϕ*[*j*] = 1 if $j\notin G_{X}$). More specifically, when platform scaling is enabled, the loss function of the Reparameterized Paired NMF Inference (Eq. 2) is updated to PoiLoss(*A*_*X*_; diag(*N*)*PQ*_*X*_) + PoiLoss(*A*_*V*_; *M*^*T*^*PQ*diag(*ϕ*)).

#### Entropy regularization

To assign the spots in *X* into distinct cell types, we optionally add an entropy regularization term of H=−ω∑(PlogP) to the loss function (2) with increasing weight *ω* = exp(*k*/*λ*) across training epochs, where *k* is the current epoch and *λ* is a hyperparameter. Lower entropy encourages each Xenium spot to be predominantly assigned to a single latent factor or cell type, rather than being evenly distributed across multiple types. In general, when the focus is imputing missing genes, we suggest a larger value of *λ* such that the Poisson losses are the dominant terms. When the focus is the deconvolution of admixed spots, we suggest a smaller value of *λ* such that entropy regularization becomes more dominant near the end of the training process. We use *λ* = 500 in simulation and deconvolution on BRCA and CRC data sets (Results, “SIID recovers the cell type expression in simulated data set”; Results, “Deconvolving cell types in cancer SRT data”) and *λ* = 1000 for imputation on BRCA and CRC data sets (Results, “Imputing missing genes in cancer SRT data”; Results, “SIID imputation leads to stromal cell subtype discovery in breast cancer Xenium data”).

#### Random restarts

As NMFs are known to be sensitive to initializations (Gaujoux and Seoighe 2010; Fathi Hafshejani and Moaberfard 2023), we always restart our model three times and use the one with the best loss value.

#### Imputing missing genes

To impute the expression of a missing or holdout gene *g* on *X*, the model is trained with $g\notin G_{X}$ and *g* ∈ *G*_*V*_. With trained parameters *P*, *Q*, and *N*, the imputed expression for *g* is diag(*N*)*PQ*_*g*_, where $Q_{g}\in R_{\geq 0}^{h}$ is the column of *Q* corresponding to *g*. Multiple genes can be imputed in the same run.

### Software availability

A PyTorch implementation of SIID is available at GitHub (https://github.com/raphael-group/siid). The repository also includes installation guides and several example Jupyter notebooks for potential users to get started. In addition, a snapshot of this repository is available as Supplemental Code.

## Supplemental Material

Supplement 1

Supplement 2

Supplement 3
