# Supplementary material for: Joint imputation and deconvolution of gene expression across spatial transcriptomics platforms
Source: Genome Res. 2025 Dec;35(12):2734–43. doi: 10.1101/gr.280555.125 (PMC12667712; doi:10.1101/gr.280555.125)
Supplement: Supplement 2 [file Supplemental_Materials.pdf]

Supplemental Methods

Contents

A Proof of the Reparameterization Lemma **2**

B Benchmarking on simulated Xenium and Visium data **2**
B.1 Steps for generating simulated Xenium and Visium data . . . 2
B.2 Benchmarking deconvolution results . . . 3
B.3 Evaluating the effect of spatial mapping matrix in SIID . . . 3
B.4 Execution time and memory usage . . . 4
B.5 Evaluation of spatially variable coverage . . . 4
B.6 Evaluation of platform scaling factor . . . 4

C Detailed benchmarking information for real data **4**
C.1 Downloading and preprocessing datasets . . . 5
C.2 Dataset statistics . . . 5
C.3 Hyperparameters of SIID . . . 6
C.4 Setup for benchmarking imputation . . . 6
C.5 Evaluating the effect of noise in spatial mapping . . . 7
C.6 Consolidated runtime information for imputation runs . . . 7
C.7 SIID identifies cell subtypes in stromal population from BRCA . . . 8
C.8 SIID with Negative Binomial Counts . . . 8

D Additional Results **9**
D.1 Reporting R^2 scores with different hyperparameters . . . 9
D.2 Reporting other correlation measures . . . 11

A Proof of the Reparameterization Lemma

To formally prove Lemma 1 we require no empty rows (rows will all zeros) in W and H. This means there are no spots with zero inferred expression, and there are no latent factors with zero inferred expression.

Lemma. Given $\overline{A_U} = WH$ where W and H has no empty rows and $W, H \geq 0$, there exists P, Q, N with $P, Q, N \geq 0, P\mathbb{1}_h = \mathbb{1}_{|S_X|}, Q\mathbb{1}_{|G_V|} = \mathbb{1}_h$ as defined in Methods - A shared cell type model between paired SRTs, such that $\overline{A_U} = \text{diag}(N)PQ$, and vice versa.

Proof. The forward direction can be proved in two steps, starting with W and H:

- Let $R_H = H\mathbb{1}_{|G_V|}$ be the row sum of H. Since H has no empty rows, $R_H > 0$ and let $W_1 = W\,\text{diag}(R_H), Q = \text{diag}(1/R_H)H$. We have $WH = W_1Q$ where Q is already row-normalized.
- Let $N = W_1\mathbb{1}_h$ be the row sum of W_1. We can further set $P = \text{diag}(1/N)W_1$ knowing $N > 0$, and since $\text{diag}(N)P = W_1$, we have $\text{diag}(N)PQ = W_1Q = WH$.

The reverse direction can be proved by setting $W = \text{diag}(N)P, H = Q$ from which we get $WH = \text{diag}(N)PQ$. □

B Benchmarking on simulated Xenium and Visium data

B.1 Steps for generating simulated Xenium and Visium data

We collect the paired scRNA-seq data, Visium data, and Xenium data from the BRCA dataset (Janesick et al. 2023) as described in Results - Setup and evaluation. Following the notation of Methods - A shared cell type model between paired SRTs the simulated Xenium slice measures genes in G_X over cell locations S_X, the simulated Visium slice measures genes in G_V over spot locations S_V, and $G_X \subset G_V$. We further cluster the cells in the scRNA-seq data and selected h distinct clusters. Our simulation process contains the following steps.

- **Step 1: Checkerboard style spatial clusters for Xenium spatial locations** We first assign each cell in X to one of h clusters. This assignment is done by dividing the coordinates of S_X into square grids, then assigning all cells in each grid to the same cluster such that that cells from adjacent grids belong to different clusters. Such assignment will create a checkerboard style (Jackson et al. 2024) pattern based on S_X. In this simulation a larger fraction of neighboring cells belong to different clusters, leading most Visium spots to contain cells from multiple cell types. Consequently state-of-the-art spatial clustering algorithms struggle to accurately deconvolve the cluster mixture proportions (Jackson et al. 2024) from this simulated data. The spatial location to cluster assignment is represented as a matrix $\widetilde{P}_X$, where $\widetilde{P}_X \in \{0, 1\}^{|S_X|\times h}$.
- **Step 2: Constructing the Poisson mean for the latent gene expression matrix** Unlike real data, in simulation we can create the Poisson mean of the latent gene expression matrix $\overline{A_U} \in \mathbb{N}^{|S_X|\times|G_V|}$. Given the scRNA-seq gene expression matrix and the cell to cluster assignments, we first compute the normalized average gene expression for each cluster, represented by $\widetilde{Q} \in \mathbb{R}^{h\times|G_V|}$, s.t. $\widetilde{Q}\mathbb{1}_{|G_V|} = \mathbb{1}_h$. Using the Xenium spatial location to cluster assignment from step 1, the simulated latent gene expression matrix is sampled from a Poisson mean $\overline{A_U} = \text{diag}\,(\widetilde{N})\widetilde{P}_X\widetilde{Q}$, where $\widetilde{N} \in \mathbb{N}^{|S_X|}$ denotes the expected gene count for each Xenium cell.
- **Step 3: Simulating Xenium gene expression matrix** The Xenium expression matrix $\widetilde{A_X} \in \mathbb{R}^{|S_X|\times|G_X|}$ is a submatrix of the latent gene expression matrix $\widetilde{A_U} \sim \text{Pois}(\overline{A_U})$ where columns are restricted to gene set G_X.
- **Step 4: Simulating Visium gene expression matrix** We use a pre-computed mapping matrix Γ to compute the Poisson mean of Visium gene expression as $\overline{A_V} = \Gamma^T\overline{A_U}$, meaning the Poisson mean for each Visium spot equals the sum of Poisson mean from each Xenium cell mapped to that spot. We then sample $\widetilde{A_V} \sim \text{Pois}(\overline{A_V})$ independently of $\widetilde{A_U}$. We additionally construct cluster mixture proportion matrix $\widetilde{P}_V \in [0, 1]^{|S_V|\times h}$ by row-normalizing $\Gamma^T\,\text{diag}\,(\widetilde{N})\widetilde{P}_X$.

Upon simulating the Xenium count matrix $\widetilde{A_X}$ and Visium count matrix $\widetilde{A_V}$, we can construct an instance $((\widetilde{A_X}, S_X), (\widetilde{A_V}, S_V), \Gamma)$ for *Reparameterized Paired NMF Inference* problem using the imputation setup described in Results - Imputation setup and holdout evaluation for one of the 10 folds. We then run SIID with h latent factors. We measure the accuracy of SIID, by comparing the estimated parameters $(\widehat{P}, \widehat{Q}, \widehat{N}, \widehat{M})$ with the ground-truth parameters as follows,

- We compute Jensen-Shannon divergence between $\widehat{P}$ and $\widetilde{P}_X$ to measure the accuracy of Xenium spot to cluster assignment.
- To evaluate the cluster assignment for Visium spots, we first calculate $\widehat{P}_V = \widehat{M}^T \widehat{P}$ using estimates from SIID. The row-normalized $\widehat{P}_V$ is compared with $\widetilde{P}_V$ as defined above in Step 4 of the simulation.
- We evaluate the gene expression for inferred clusters by comparing $\widetilde{Q}$ to $\widehat{Q}$. Here we compute the pairwise Pearson correlation coefficients between the rows of $\widetilde{Q}$, and $\widehat{Q}$.
- We also evaluate the performance of our model in imputing holdout genes in Xenium panel by the same process described in Results - Imputation setup and holdout evaluation.

B.2 Benchmarking deconvolution results

STdeconvolve setup

To do a fair comparison with SIID, we run STdeconvolve on Visium data on the shared genes between Xenium and Visium data. As the remaining genes are low in number we did not perform any gene selection before running STdeconvolve . The other steps are same as described in STdeconvolve documentation [3].

SpiceMix setup

We follow instructions given in the SpiceMix paper (Chidester et al. 2023) and SpiceMix GitHub repository[4] and preprocess the Visium dataset as follows. First, all genes that are expressed in less than 10% of spots are removed. Second, all counts are log-normalized to a target UMI count of 10,000 per cell. Third, the neighbourhood graph is derived by connecting each spot with its 6 closest neighbors. Afther these transformations, SpiceMix is run with the default parameters for 200 epochs, with the number of metagenes set to the number of hidden dimensions used in SIID and STdeconvolve .

Computing cell type proportion matrix

We first map latent factors inferred from SIID to true cell types gene expressions to find the best mapping between the latent factors and the cell types by a greedy match process. Given that correspondence we rearrange the columns of mixture proportion matrix P to match the true cell type proportions. A similar approach is used to find the best order for cluster mixture proportions from STdeconvolve . Finally, we use Jensen-Shannon divergence (Menéndez et al. 1997) (JS divergence) to evaluate the quality of predicted cell type mixture proportions when compared to the true mixture proportions. A lower JS divergence signifies that the predicted mixture proportion is closer to the ground truth.

B.3 Evaluating the effect of spatial mapping matrix in SIID

B.3.1 Effect of noisy spatial mapping matrix

We evaluate the reliability of spatial mapping in the resulting imputation and deconvolution performance by introducing noise to the mapping matrix Γ. To control the level of noise, we randomly select τ percent of aligned spot pairs, i.e. entries (i, j) where $\Gamma[i, j] = 1$, and swap with a Xenium-to-Visium spot pair (i', j') such that $\Gamma(i', j') = 0$, resulting in the noisy spatial mapping matrix Γ' where $\Gamma'[i', j'] = 1$, and $\Gamma'[i, j] = 0$. In simulation we vary $\tau \in \{0, 10, 20, \ldots 100\}$ where $\tau = 0$ means $\Gamma' = \Gamma$, and $\tau = 100$ means all the spatial mappings are swapped in Γ'. We observe SIID yields comparable R^2 scores with different levels of noise introduced to Γ (Supplemental Figure S2). However the JS divergence, which measures the deconvolution performance for the Visium spots, increases as noise in the spatial mapping rises, indicating poorer deconvolution performance as the spatial mapping matrix becomes less reliable.

[3] https://github.com/JEFworks-Lab/STdeconvolve
[4] https://github.com/ma-compbio/spicemix

B.3.2 Effect of non-rigid transformation on SIID

To assess the effect of physical distortion that occurs during sample preparation in spatial transcriptomics, we performed an additional simulation where we warped and rotated the coordinates of the Xenium slice to create a simulated Visium slice. Specifically, we applied a sinusoidal distortion to 2D coordinates, where the horizontal displacement follows $x' = x + A_x \sin(2\pi y/\lambda_y)$ and the vertical displacement follows $y' = y + A_y \cos(2\pi x/\lambda_x)$. The parameters $A_x = 30$ and $A_y = 20$ control the amplitude of distortion in each direction, while $\lambda_x = 200$ and $\lambda_y = 200$ determine the spatial wavelengths of the oscillations. This creates a bidirectional wave-like deformation, where points are displaced horizontally based on their y-coordinate with period λ_y, and displaced vertically based on their x-coordinate with period λ_x, resulting in a sinusoidal grid distortion with intersecting wave patterns. After such a transformation, we applied a rotation of θ of 30 degrees (see Supplemental Figure S4A).

We recompute Γ_w based on the transformed spatial coordinates. We ran SIID using Γ_w and compared to the results obtained using the unaltered spatial mapping matrix Γ. We compared the three reconstructed matrices — namely, the cell type profile matrix Q, the Xenium spot to cell type assignment matrix P_X, and the Visium spot to cell type assignment matrix P_V — with their corresponding ground truth. We performed 10-fold cross validation using 90% of the Xenium genes for training and using the remaining 10% for testing. Specifically, we compared the row-wise correlation coefficient of each matrix: for Q, this reflects accurate reconstruction of the cell type profile, and similarly for P_X and P_V, this reflects accurate reconstruction of the cell type assignment for each Xenium spots and Visium spots, respectively. We observe that the use of misaligned spatial mapping matrix Γ_w does not alter the performance for reconstructing the Q and P_X matrices, indicating robustness of those reconstructions to spatial misalignment. However, we observe a marked drop in performance for the P_V matrix reconstruction, indicating the poor reconstruction of the cell type proportion in the Visium spots across all ten folds (Supplemental Figure S4B).

B.4 Execution time and memory usage

We measure the time and memory usage for running SIID and Tangram by profiling the execution of both the tools using Python's `time` and `tracemalloc` modules. We note that the recorded time and peak memory usage for different simulation configurations remain relatively stable across different levels of coverage, grid sizes, and cluster counts, indicating that computational costs are scalable across these parameters. While SIID requires more execution time than Tangram , Tangram requires significantly higher memory (Supplemental Figure S7).

B.5 Evaluation of spatially variable coverage

We evaluated the imputation performance of SIID on a simulated dataset (with $l = 10, h = 8$) and with the coverage ρ varying across space (SIID recovers the cell type expression in simulated dataset). Specifically, we set $\rho(x) = \frac{x - x_{\min}}{x_{\max} - x_{\min}}$ to be a function of the x-coordinate of the Xenium spot (Supplemental Figure S3A). For both SIID and Tangram , the overall R^2 score using spatially variable coverage $\rho(x)$ is slightly higher (Supplemental Figure S3B) than the R^2 score attained with a constant coverage ($\rho = 0.5$).

B.6 Evaluation of platform scaling factor

We evaluate the effect of platform scaling in the performance of SIID and Tangram . We use platform scaling while simulating the Xenium and Visium datasets by scaling the expression of each gene by a gene-specific factor that is constant across all cells. To emulate the real data, we first measure the platform scaling factors for the shared genes between the BRCA Xenium and Visium data (Supplemental Figure S11A). Later, we sample from this empirical distribution of platform scaling factors to simulate the gene expression of the Visium data. While running SIID, we enable platform scaling (Implementation, parameter inference and imputing missing genes). We observe that SIID achieves a superior R^2 score (Supplemental Figure S11B) as compared to Tangram in imputing gene expression for the holdout genes in Xenium dataset.

C Detailed benchmarking information for real data

In this section, we describe detailed setups for running our method and other benchmarked methods over real data.

C.1 Downloading and preprocessing datasets

BRCA pair. We downloaded the following datasets from 10x Genomics website[5]: Xenium In Situ Sample 1, Replicate 1; Visium Spatial; FRP (Fixed RNA Profiling) as the scRNA-seq pairing. We also download the cell type annotation for FRP and Xenium in the same page.

CRC pair. We downloaded the following datasets from 10x Genomics website[6]: Visium CytAssist V2, Sample P2 CRC; Xenium In Situ, Sample P2 CRC; and Chromium Single Cell Flex, aggregated (as the scRNA-seq pairing). The aggregated Chromium dataset contains scRNA-seq data from 8 different samples, and we subset the it to only cells collected from the matching Sample P2 CRC (indices 1, 3, 5 and 7).

Slice Alignment. For the BRCA pair, we follow the suggestion in the data paper (Janesick et al. 2023) and manually annotate 5 pairs of key points on both slices, then compute a rigid transformation minimizing distance between these key point pairs (also known as Orthogonal Procrustes problem) (Gower and Dijksterhuis 2004). For the CRC pair, we run PASTE2 (Liu et al. 2023) with certain changes. Specifically, we create a grid of 60μm*60μm over the original Xenium slice roughly matching the size of Visium spots (65um hexagonal). For each 60μm*60μm tile in the slice, we convert all cells in this tile to a Xenium pseudo-spot. The gene expression of this pseudo-spot is the sum of all covered cells, and its spatial location is the weighted center of mass (by total UMI counts). Next, we run PASTE2 to align the Visium slice with the converted Xenium pseudo-slice and compute a rigid transformation by solving the Procrustes problem minimizing the distance of each Visium spot to the barycenter in Xenium coordinate.

Caveats. There are both biological and technical sources of variation between adjacent slices that complicate the alignment. Biologically, the physical distance (often tens of microns) between adjacent tissue sections can lead to substantial differences in transcriptional profiles at the corresponding spatial locations in each slice, particularly in tissue regions with spatially heterogeneous cell types. In this scenario, recovering the true alignment between the slices is extremely difficult. Technically, misalignment can arise from a range of artifacts during sample preparation, mounting and imaging. For example, tissue sections can be shredded, torn, stretched or squashed, or may naturally degrade during processing. Additionally, SRT technologies are not perfect and can introduce significant noise and bias during data acquisition. Our experiments with non-rigid transformations and rotations presented in Supplemental Methods B.3 and C.5 are representative of such realistic misalignment scenarios.

C.2 Dataset statistics

Supplemental Table S1 contains detailed statistics regarding the data used for benchmarking. The rows indicated as (in-Xenium-panel) are the statistics for Visium dataset when restricted to paired Xenium panel.

Datasets	Component	No. of Spots	Panel Size	Total Counts	Count/Gene	Count/Spot
BRCA	Xenium	167,780	313	32,102,956	102,565	191
	Visium	4,992	(WT)	115,297,808	6,375	23,096
	(in-Xenium-panel)	*4,992*	*307*	*4,123,612*	*13,431*	*826*
	scRNA-seq	30,365	(WT)	204,904,784	11,331	6,748
CRC	Xenium	340,837	422	42,073,224	99,699	123
	Visium	4,269	(WT)	404,804,672	22,383	94,824
	(in-Xenium-panel)	*4,269*	*408*	*18,012,420*	*44,148*	*4,219*
	scRNA-seq	41,993	(WT)	149,924,208	8,291	3,575

Table S1: Dataset statistics. *(in-Xenium-panel)* denotes Visium dataset subsetted to matching Xenium gene panel. WT stands for whole-transcriptomic (18 thousand genes).

[5] https://www.10xgenomics.com/products/xenium-in-situ/preview-dataset-human-breast
[6] https://www.10xgenomics.com/products/visium-hd-spatial-gene-expression/dataset-human-crc

C.3 Hyperparameters of SIID

SIID contains a number of hyperparameters. Most of these parameters are chosen based on common sense and a small-scale parameter search.

- Number of latent factors h determines the size of P and Q of the factorization, and is loosely based on how many cell types the datasets have. In simulation, h is set to match the data generation process, and in real data, it is set to 20 or 40 for `BRCA` and `CRC` respectively. This is the only dataset-specific parameter we employ.
- We described platform scaling technique in Implementation, parameter inference and imputing missing genes as a way to model platform-specific count modeling between Xenium and Visium datasets. This is disabled for simulation both in dataset generation and inference, and is enabled when running with real data.
- SIID uses entropy regularization (Implementation, parameter inference and imputing missing genes) to promote assigning Xenium cells to smaller number of latent factors. With fixed total of 5,000 training epochs and regularization weight $\exp(k/\lambda)$ where n_{epoch} is the epoch number, higher λ means weaker regularization strength. We use $\lambda = 1000$ for real data imputation (Imputing missing genes in cancer SRT data), and $\lambda = 500$ elsewhere.
- SIID has the flexibility of using genes inside and outside the targeted panel during inference. For our runs, we choose to not use Visium genes outside Xenium panel (except those in holdout set for imputation).
- SIID is run with 3 restarts, and the run with lowest total loss is used for prediction. It also uses a ℓ_2 regularization over raw model parameters (Implementation, parameter inference and imputing missing genes), whose weight is set to 10^{-5} for all runs. The model is optimized with Adam optimizer with learning rate of 0.05.

We also benchmark performance of SIID under different hyperparameter choices. Specifically, for both `BRCA` and `CRC` datasets, we test combinations of the following parameter values:

- Number of latent factors h is chosen from (5, 10, 20, 30, 40, 50).
- The entropy regularization strength λ is chosen from (300, 500, 1000, 1500, 2000) for both datasets. The number of training epochs are fixed at 5000.
- The number of random restarts being 1 or 3.

We present the results for these control experiments in Supplemental Methods D.1.

C.4 Setup for benchmarking imputation

We select the methods to benchmark by the top performers in (Li et al. 2022), excluding SpaOTsc (Cang and Nie 2020) as it requires a pairwise distance matrix over Xenium data with over 100K cells, and excluding SpaGE (Abdelaal et al. 2020) as it operates over log-normalized data while we benchmark imputation with raw counts. We additionally select SLAT (Xia et al. 2023) and SANTO (Li et al. 2024) as representatives of SRT-alignment-based imputation methods.

- Tangram (Biancalani et al. 2021) is run with Visium dataset as the single-cell data, and the Xenium dataset as the spatial data with default parameter and 1000 training epoches. We also run Tangram in two configurations, once in default mode (cell mode), and once in cluster mode with cluster labels generated by running Leiden clustering (Traag et al. 2019) in scanpy package (Wolf et al. 2018) with default parameters. Tangram with scRNA-seq pairing is run in the same way, except that we downsample the scRNA-seq dataset for `CRC` by 2× when Tangram is run in cell mode. This is so it could finish in a reasonable amount of time.
- gimVI (Lopez et al. 2019) is run in default parameters with the same number of latent factors as SIID, and optimized for 200 epochs. As advised by authors, We also remove empty cell or spots before optimization. However, we could not finish optimization due to model parameters becoming `NaN` frequently in the middle of the optimization after a large number of attempts and trying over many different configurations.
- SLAT (Xia et al. 2023) is run with parameters recommended for Xenium-Visium pairing (120 neighbors for building kNN graph for Xenium, 5 neighbors for building kNN graph for Visium). For each Xenium cell (even outside of Visium-Xenium overlap area), SLAT matches it to a Visium spot. Imputation of a holdout gene is performed by copying the expression of such holdout gene on matched Visium spot to the Xenium cells.

- SANTO (Li et al. 2024) proposes an imputation strategy from two aligned SRT slices based on Poisson regression, which we re-implement and benchmark. For each holdout gene g, we train a Poisson regression model of form $\Gamma_I V_g \sim \text{Pois}(A_{XI} D)$; here, I denotes the set of Xenium cell that maps to a Visium spot (in the intersecting area of Visium and Xenium slices), A_{XI} and Γ_I denote A_X and Γ subseted to I respectively, V_g denotes the Visium expression for the holdout gene, and $D \in \mathbb{R}^{|G_X|}$ is the combination coefficients for imputing the holdout gene. We use the alignment as described in Setup and evaluation, and identical mapping Γ as used in SIID. We impute the expression for all Xenium cells (inside or outside I) after training and evaluate correlation on all cells.
- For Baseline A, the estimated expression for a Xenium gene g is given by ΓV_g, where V_g refer to the expression of gene g in Visium. Intuitively, the Visium spot expression is copied to each Xenium cell mapped to that spot through Γ. Xenium cells that are not mapped to a Visium spot (as the datasets are partially aligned) are assigned zero expression.
- For Baseline B, let $C_X = A_X \mathbb{1}_{|G_X|}$ be the observed total count per Xenium cell. If a Xenium cell i is mapped to Visium spot j, we let $w[i] = \frac{C_X[i]}{\sum_{k:\Gamma[k,j]=1} C_X[k]}$ be the weight of cell i among all cells mapped to the same spot. The estimate for a Xenium gene g is then given by computing the estimate for Baseline A, then scale by $w[i]$ for each cell i. Intuitively, this means the counts in Visium spot j is distributed among Xenium cells mapped to that spot proportional to observed Xenium counts.
- For Baseline C, from the outputs of Baseline A, we apply a kNN smoothing where the expression of each Xenium cell is calculated as the average of its 120 nearest neighbors. We then follow the partition-scaling steps as described in Baseline B.
- For Baseline D, from the outputs of Baseline A, we first perform the partition-scaling as done in Baseline B, then apply kNN smoothing as described above.

C.5 Evaluating the effect of noise in spatial mapping

We follow the identical setup as in Supplemental Methods B.3 to evaluate the effect of changing Γ in imputation performance. See Supplemental Figure S2C and Supplemental Figure S2D for their effect on imputing real datasets.

We also evaluated the consequences of distortion of the mapping matrix Γ on both datasets. For this test, we run SIID with identical hyperparameters, but rotate the Visium slice clockwise around its centroid from 10 to 90 degrees in increments of 10. Larger rotation means most Xenium cells are mapped to more distant Visium spots. We observe that SIID's performance decreases as the angle of rotation increases, but that overall performance does not degrade substantially (Supplemental Figure S6).

C.6 Consolidated runtime information for imputation runs

SIID are benchmarked on a single Tesla P100 GPU with 12GB memory, and most other methods (if CPU-Bound) are run on a cluster with 20 Xeon E5-2690 CPUs. SIID is run five times per fold with 3 restarts each and the reported values in Table 1 and Supplemental Table S2 are averaged across 5 runs. Average runtime for the benchmarked methods for each fold in seconds can be found in Supplemental Table S2, including Visium pairing and scRNA-seq pairing. Without the 2× downsamping in CRC scRNA-seq dataset, Tangram cannot finish within 2 days or 172,800 seconds. For Tangram in cluster mode, we do not include the time taken for running Leiden clusters.

		Tangram (Visium)				Tangram (scRNA-seq)	
Dataset	SIID (3 runs)	cell mode	cluster mode	SLAT	SANTO	cell mode	cluster mode
BRCA	403s	8,632s	< 300s	< 300s	4,020s	43,309s	< 300s
CRC	965s	17,232s	< 300s	429s	12,814s	65,240s	< 300s

Table S2: Comparison of average run time in seconds per fold for the benchmarked methods.

We note that Tangram in cell mode for BRCA and CRC datasets takes 2.5 and 5 hours to complete (Supplemental Table S2), in part because running the Tangram model on GPUs requires a large amount of GPU memory to store the full $|S_X|\times|S_V|$ entries of the mapping matrix M. In contrast, SIID stores only $h(|S_X|+|G_V|) \approx h|S_X|$ (since $|S_X| \gg |G_V|$) parameters for P and Q, around 100× fewer than Tangram for our benchmarking datasets.

To further evaluate scalability of SIID, we benchmark the time and peak GPU memory for varying sizes of input and latent space. Since most work is done on the GPU, CPU memory is not a constraint for SIID. Specifically, we ran SIID on the BRCA dataset in the following settings:

- Setting h, the number of latent factors to 20, 40, 60, 80 and 100.
- Setting the number $|G_V|$ of Visium genes in inference to 400, 800, 1200, 1600, 2000.
- More Xenium cells: We make between 1 and 5 copies of the Xenium cells. The copies have their spatial location shifted by $N(0, 10)$ in both dimensions and gene count randomized by a Poisson distribution.
- More Visium spots (roughly corresponding to higher grid resolution): Similar to the above experiment, but location shifts are 5 times larger.

As shown in Supplemental Figure S5, SIID takes up to 20% more time and GPU memory with up to 5× of latent factors, more genes, or larger Visium dataset. However, the resource usage scales roughly linearly as the size of the Xenium dataset. This is consistent with our proposed theory that the size of the Xenium dataset $|S_X||G_X|$ is the dominating factor in evaluating the time and memory usage of SIID.

C.7 SIID identifies cell subtypes in stromal population from BRCA

We start with the paired single-cell RNA-seq dataset (Supplemental Figure S13A) and perform an unsupervised clustering followed by delineating marker genes for each unsupervised cluster (Supplemental Figure S13B). We note that many of these differentially expressed genes are not present in the Xenium pre-defined panel (marked in red) which in turn makes it impossible to delineate cell subtypes of this compartment. To select the set of candidate genes for imputation, we followed the standard procedure implemented in the `scanpy` (Wolf et al. 2018) package. Specifically, we identified highly variable genes by calculating gene-wise statistics and then selecting those that met the criteria of having a mean expression between 0.0125 and 3, and a dispersion (variability) greater than 0.5. This procedure produces 3,000 highly variable genes out of around 18,000. We next run SIID to impute the expression of these genes from the companion Visium dataset. The stromal cells (Supplemental Figure S13C) in the imputed Xenium dataset is further sub clustered into four subgroups (Supplemental Figure S13D).

We further analyzed the spatial co-localization of the Stromal and T-cell hybrid cell type with stromal cell sub types annotated by SIID in BRCA Xenium data. After overlaying the Stromal–T cell hybrid spots with stromal subtypes on the Xenium spatial map (Supplemental Figure S14), we observed that these cell types share spatial neighborhoods. To quantify this, we computed the spatial co-occurrence between Stromal-T cell hybrid cells and Stromal subtypes using Squidpy's `squidpy.gr.co_occurrence`[7](Supplemental Figure S14B). Across various inter-spot distances, all stromal subtypes—except adipocyte-rich stroma—showed comparable co-occurrence profiles. In the imputed Xenium data, we further examined genes from the immune–stromal niche signature and found them expressed in the Stroma–T cell hybrid cluster as well (Supplemental Figure S14C), suggesting that spots from both cell types share an overlapping gene-expression program.

C.8 SIID with Negative Binomial Counts

Modeling. Inspired by gimVI (Lopez et al. 2019), we implement an alternative model where we assume that Xenium counts follow a Poisson distribution, while Visium counts follow a negative binomial distribution. The negative binomial mean $\overline{A_V} = M^T PQ$ is the same as in the count-scaled reparameterization described in A shared cell type model between paired SRTs. The overdispersion α_V is gene-specific but consistent between spots. Formally, the loss function is

$$\text{PoiLoss}(A_X; \text{diag}(N)PQ_X) + \text{NegBinomLoss}(A_V; M^T PQ, \mathbf{1}_{|S_V|}\alpha_V^T)$$

[7] https://squidpy.readthedocs.io/en/stable/api/squidpy.gr.co_occurrence.html

Here, $\alpha_V \in \mathbb{R}_+^{|G_V|}$ is the overdispersion for each gene, and the matrix $\mathbf{1}_{|S_V|}\alpha_V^T$ indicates that all spots for the same gene share the same overdispersion parameter. For a given spot-gene pair, with observed count X, inferred mean m and inferred verdispersion α, the loss function (negative log-likelihood) is defined as:

$$\begin{aligned}\text{NegBinomLoss}(X \mid m, \alpha) &= \text{NegBinomNLL}(X \mid p, r)\\ &= -\ln \Gamma(X + r) + \ln \Gamma(r) + \ln(X!) - r\ln(p) - X\ln(1-p)\\ &= -\ln \Gamma(X + 1/\alpha) + \ln \Gamma(1/\alpha) + \ln(X!) + \ln(1+\alpha m)/\alpha + X\ln(1 + 1/\alpha m)\end{aligned}$$

where $r = 1/\alpha$ and $p = 1/(1 + \alpha m)$ are the parameters of the negative binomial in its natural parameterization, and Γ is the Gamma function that extends factorial to complex numbers.

Implementation. We implement this version of SIID, optimizing α_V alongside other parameters such as P, Q and M. Since overdispersion cannot be negative, we similarly perform gradient descent on $\log \alpha_V$ instead.

Evaluation. We evaluate this version of SIID on two real datasets in the identical setup as described in Imputing missing genes in cancer SRT data. We obtain similar performance on BRCA dataset (average gene-wise R^2 of 0.2498, compared to 0.2527 of original version) and slightly worse on CRC dataset (average gene-wise R^2 of 0.2013, compared to 0.2248 of the original version). Evaluation on other metrics can be found in Supplemental Table S6 under the column "NegBinom".

D Additional Results

D.1 Reporting R^2 scores with different hyperparameters

As outline in the second half of Supplemental Methods C.4, we benchmark SIID on two real datasets with varying hyperparameters. The final configuration used elsewhere is highlighted in bold.

		Entropy Regularization Strength				
Latent Factors	Restarts	$\lambda = 300$	$\lambda = 500$	$\lambda = 1000$	$\lambda = 1500$	$\lambda = 2000$
$h = 5$	–	0.1663	0.1739	0.1850	0.1835	0.1824
	3	0.1668	0.1706	0.1897	0.1875	0.1879
$h = 10$	–	0.1821	0.2075	0.2313	0.2298	0.2298
	3	0.1911	0.2068	0.2309	0.2317	0.2298
$h = 20$	–	0.1484	0.1689	0.2477	0.2503	0.2490
	3	0.1454	0.1546	**0.2527**	0.2536	0.2551
$h = 30$	–	0.1145	0.1312	0.2530	0.2495	0.2469
	3	0.1210	0.1243	0.2604	0.2568	0.2529
$h = 40$	–	0.1001	0.1241	0.2386	0.2412	0.2393
	3	0.1002	0.1236	0.2457	0.2439	0.2401
$h = 50$	–	0.0902	0.1178	0.2269	0.2250	0.2246
	3	0.0928	0.1179	0.2329	0.2376	0.2308

Table S3: Mean holdout R^2 scores for SIID on the BRCA dataset with varying parameters.

		Entropy Regularization Strength				
Latent Factors	Restarts	$\lambda = 300$	$\lambda = 500$	$\lambda = 1000$	$\lambda = 1500$	$\lambda = 2000$
$h = 5$	–	0.1401	0.1423	0.1513	0.1513	0.1504
	3	0.1423	0.1444	0.1522	0.1529	0.1542
$h = 10$	–	0.1575	0.1620	0.1786	0.1774	0.1776
	3	0.1602	0.1649	0.1792	0.1795	0.1792
$h = 20$	–	0.1674	0.1770	0.2116	0.2125	0.2119
	3	0.1678	0.1775	0.2125	0.2143	0.2129
$h = 30$	–	0.1655	0.1753	0.2207	0.2198	0.2210
	3	0.1680	0.1804	0.2224	0.2225	0.2227
$h = 40$	–	0.1541	0.1700	0.2209	0.2206	0.2227
	3	0.1576	0.1713	**0.2248**	0.2229	0.2255
$h = 50$	–	0.1353	0.1479	0.2188	0.2168	0.2188
	3	0.1359	0.1515	0.2217	0.2221	0.2228

Table S4: Mean holdout R^2 scores for SIID on the CRC dataset with varying parameters.

D.2 Reporting other correlation measures

We selected R^2 as our primary metric due to its use in previous studies on gene imputation (Biancalani et al. 2021). Additionally, we note that the values imputed by SIID (or Tangram) are not integer counts, making count-based correlation measures not directly applicable either. To strengthen the conclusions of the manuscript, in Supplemental Table S5, we further evaluate SIID by four other metrics as suggested and detailed in a recent benchmarking paper (Li et al. 2022) – Pearson cross correlation (PCC), structural similarity index measure (SSIM), root mean square error (RMSE), and Jensen-Shannon (JS) divergence. As shown in Supplemental Table S5, SIID has the best performance by all listed measures.

			Tangram			
Metric	Dataset	SIID	cell mode	cluster mode	SLAT	SANTO
R^2 ↑	BRCA	**0.2527**	0.1874	0.1557	0.0688	0.1042
	CRC	**0.2248**	0.1789	0.1382	0.0608	0.0727
PCC ↑	BRCA	**0.4470**	0.3789	0.3402	0.2015	0.2620
	CRC	**0.4099**	0.3644	0.2995	0.1997	0.2236
SSIM ↑	BRCA	**0.3456**	0.3112	0.1904	0.1192	0.2239
	CRC	**0.4191**	0.3905	0.2761	0.1017	0.3437
RMSE ↓	BRCA	**1.0261**	1.0952	1.1317	1.2542	1.2027
	CRC	**1.0609**	1.1089	1.1658	1.2596	1.2399
JS ↓	BRCA	**7.1959**	8.3165	8.3599	12.4704	8.9354
	CRC	**8.2665**	9.1641	9.2956	12.4493	9.8258

Table S5: Comparison of selected methods for imputing Xenium gene expression from paired Visium data using different metrics. The arrow following the metric name indicates whether higher is better (↑) or lower is better (↓). Bold indicates best performance in each metric and dataset pair.

With results from Tangram paired with scRNA-seq as well as SIID with Negative Binomial counts, SIID with Poisson counts model has the best performance in all but one measure where it's second best.

		SIID		Tangram (Visium)				Tangram (scRNA-seq)	
Metric	Dataset	Poisson	NegBinom	cell	cluster	SLAT	SANTO	cell	cluster
R^2 ↑	BRCA	**0.2527**	0.2498	0.1874	0.1557	0.0688	0.1042	0.2194	0.2203
	CRC	**0.2248**	0.2013	0.1789	0.1382	0.0608	0.0727	0.1960	0.1823
PCC ↑	BRCA	**0.4470**	0.4431	0.3789	0.3402	0.2015	0.2620	0.4126	0.4176
	CRC	**0.4099**	0.3786	0.3644	0.2995	0.1997	0.2236	0.3794	0.3700
SSIM ↑	BRCA	0.3456	0.3429	0.3112	0.1904	0.1192	0.2239	**0.4930**	0.2994
	CRC	0.4191	0.3953	0.3905	0.2761	0.1017	0.3437	**0.5786**	0.3494
RMSE ↓	BRCA	**1.0261**	1.0296	1.0952	1.1317	1.2542	1.2027	1.0609	1.0575
	CRC	**1.0609**	1.0900	1.1089	1.1658	1.2596	1.2399	1.0926	1.1038
JS ↓	BRCA	**7.1959**	7.2051	8.3165	8.3599	12.4704	8.9354	7.8144	7.8562
	CRC	**8.2665**	8.4766	9.1641	9.2956	12.4493	9.8258	8.8543	8.8650

Table S6: Similar to Supplemental Table S5, with scRNA-seq pairing results from Tangram and SIID with Negative Binomial counts (Supplemental Methods C.8) also reported. The arrow following the metric name indicates whether higher is better (↑) or lower is better (↓). Bold indicates best performance in each metric and dataset pair.
