# Supplementary material for: Joint imputation and deconvolution of gene expression across spatial transcriptomics platforms
Source: Genome Res. 2025 Dec;35(12):2734–43. doi: 10.1101/gr.280555.125 (PMC12667712; doi:10.1101/gr.280555.125)
Supplement: Supplement 3 [file Supplemental_Figures.pdf]

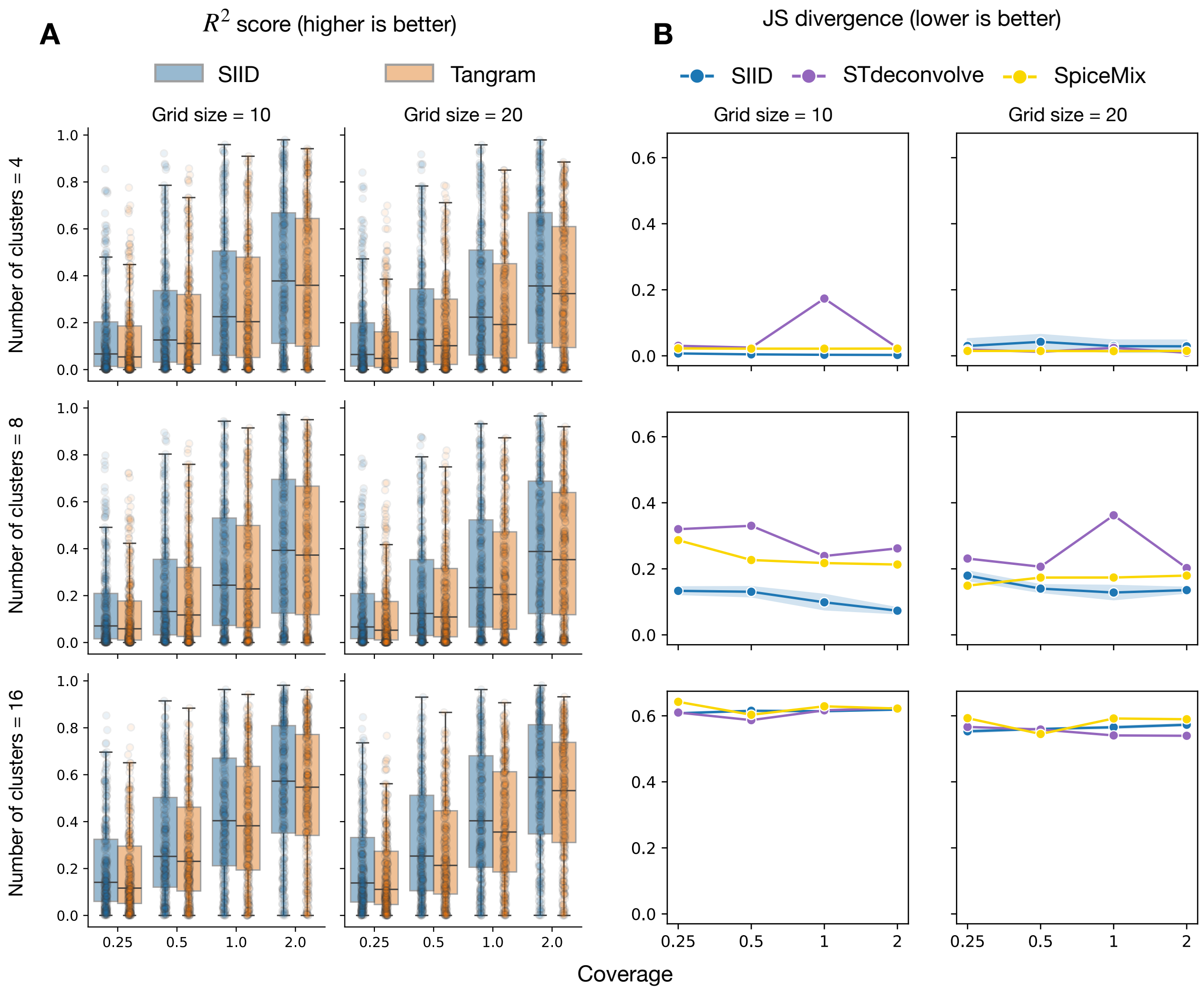

Figure S1: **(A)** R^2 score for each gene's expression between predicted values (from SIID and Tangram) and ground truth across different simulation settings. **(B)** Jensen-Shannon (JS) divergence of predicted cell type mixture proportions compared to the ground truth for SIID, STdeconvolve and SpiceMix .

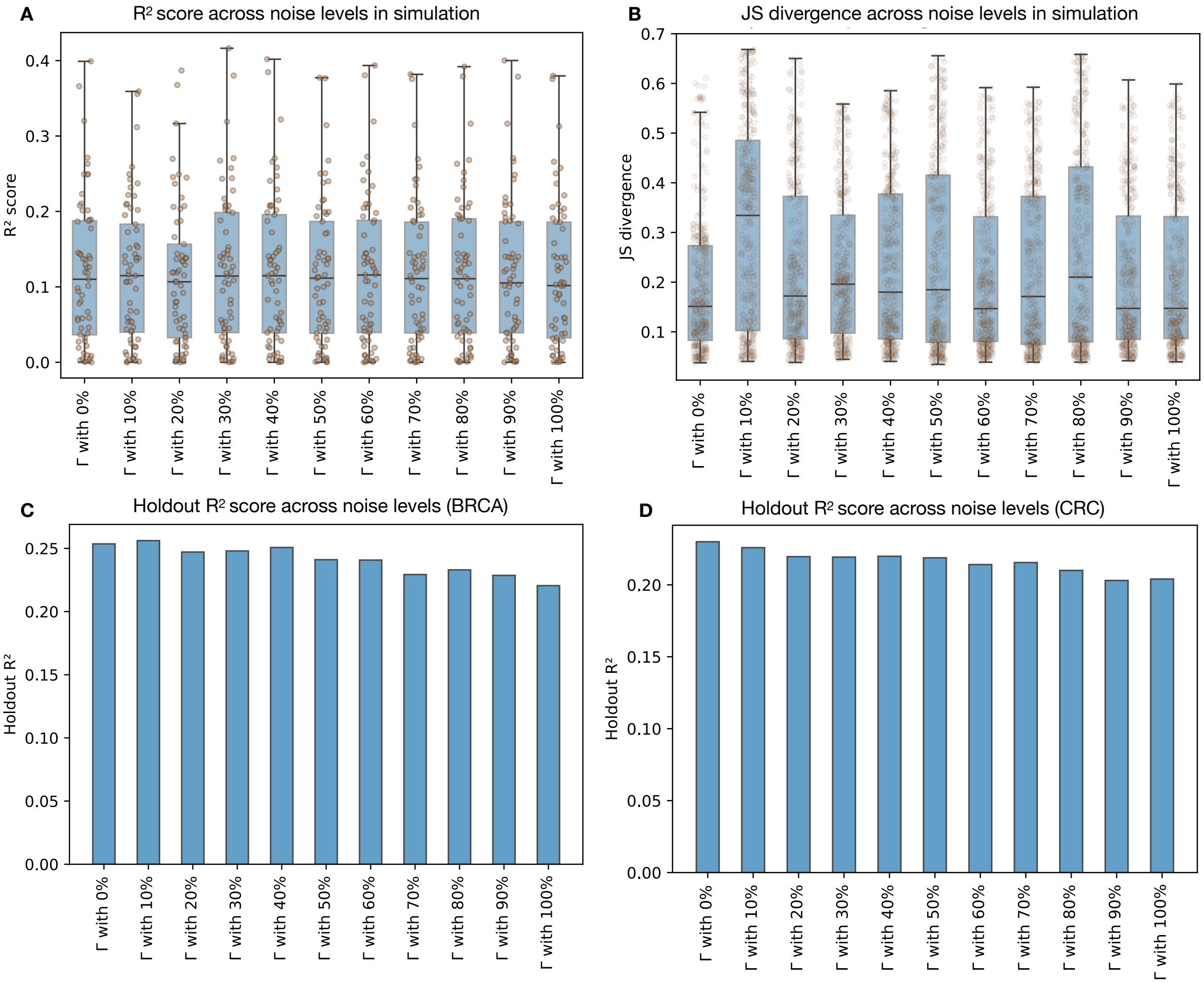

Figure S2: Evaluating model performance in the presence of perturbed Γ. **(A)** R^2 score of imputed holdout genes across different level of noise in simulated scenarios. Each point indicate a gene. **(B)** JS divergence across Visium spots across different level of noise in simulated scenarios. Each point indicate a Visium spot in simulation. **(C)** Average R^2 score of imputed holdout genes across different level of noise in BRCA holdout experiments. **(D)** Same as panel C over the CRC dataset.

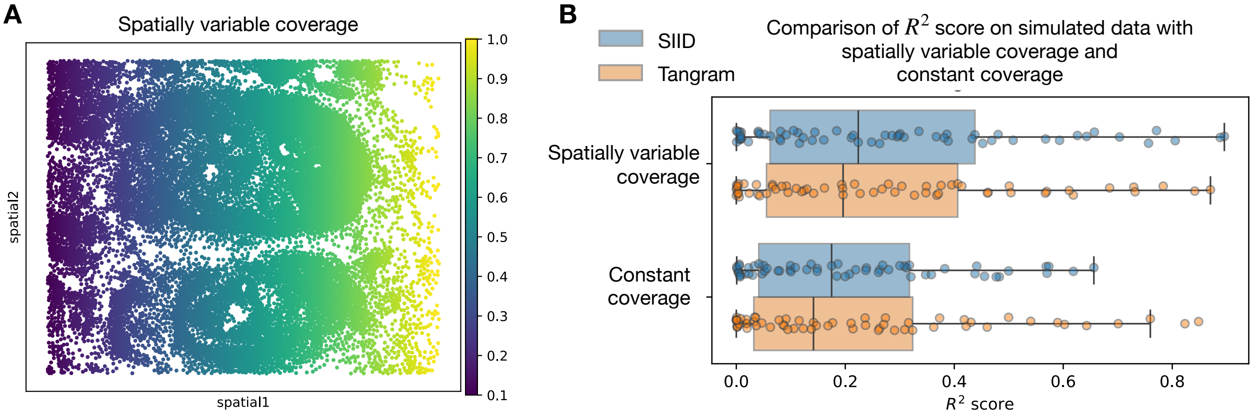

Figure S3: Evaluating the effect of spatially varying coverage in simulation. **(A)** Visualization of spatially varying coverage in simulated Xenium data. **(B)** Comparing R^2 scores of SIID and Tangram on simulated data with spatially varying coverage vs constant coverage.

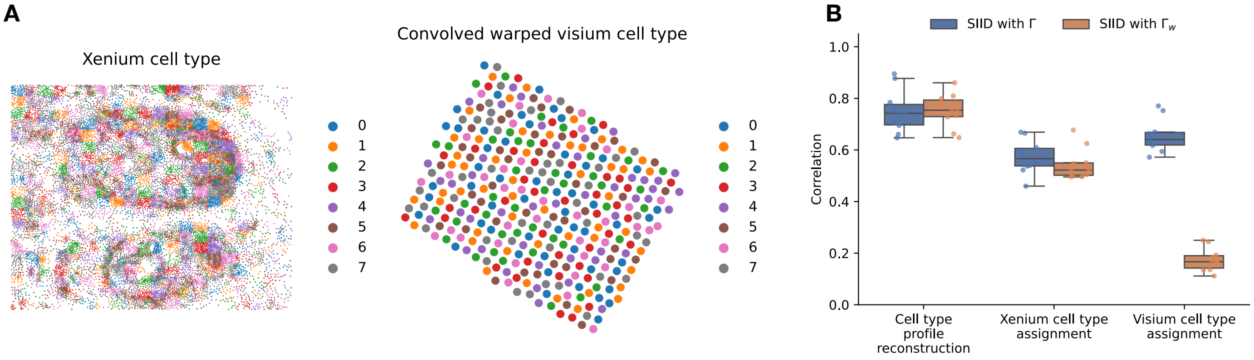

Figure S4: Evaluating the effect of non-rigid transformation. **(A)** Sinusoidal grid distortion and subsequent rotation on the Visium coordinates to create warped coordinates. **(B)** Row-wise correlation coefficient with cell type profile matrix Q, Xenium spot to cell type assignment P_X, and Visium spot to cell type assignment matrix P_V.

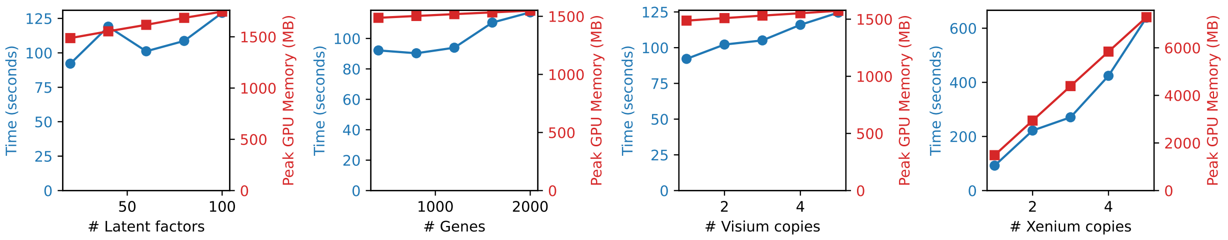

Figure S5: Time (left axis, blue) and peak GPU memory (right axis, red) used for SIID in varying configurations, based on a real run over the BRCA dataset. From left to right, the number of latent factors, number of total genes, size of Visium dataset and size of Xenium dataset are scaled up to 5 times.

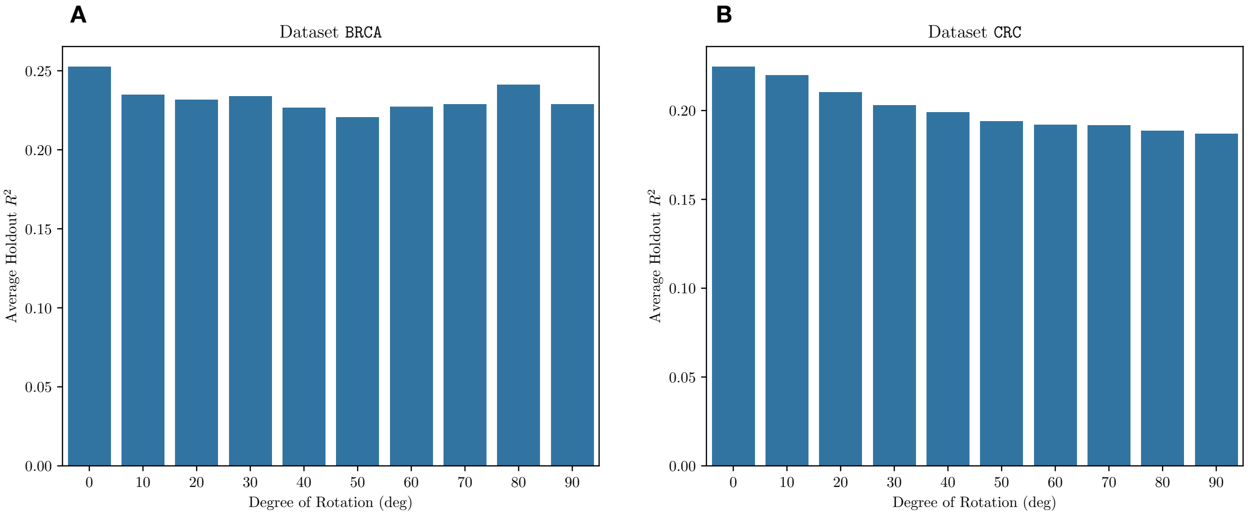

Figure S6: Evaluating model performance in the presence of misalignment Γ in real data. **(A)** R^2 score of imputed holdout genes across different degree of rotation in the BRCA dataset. **(B)** Same as panel A over the CRC dataset.

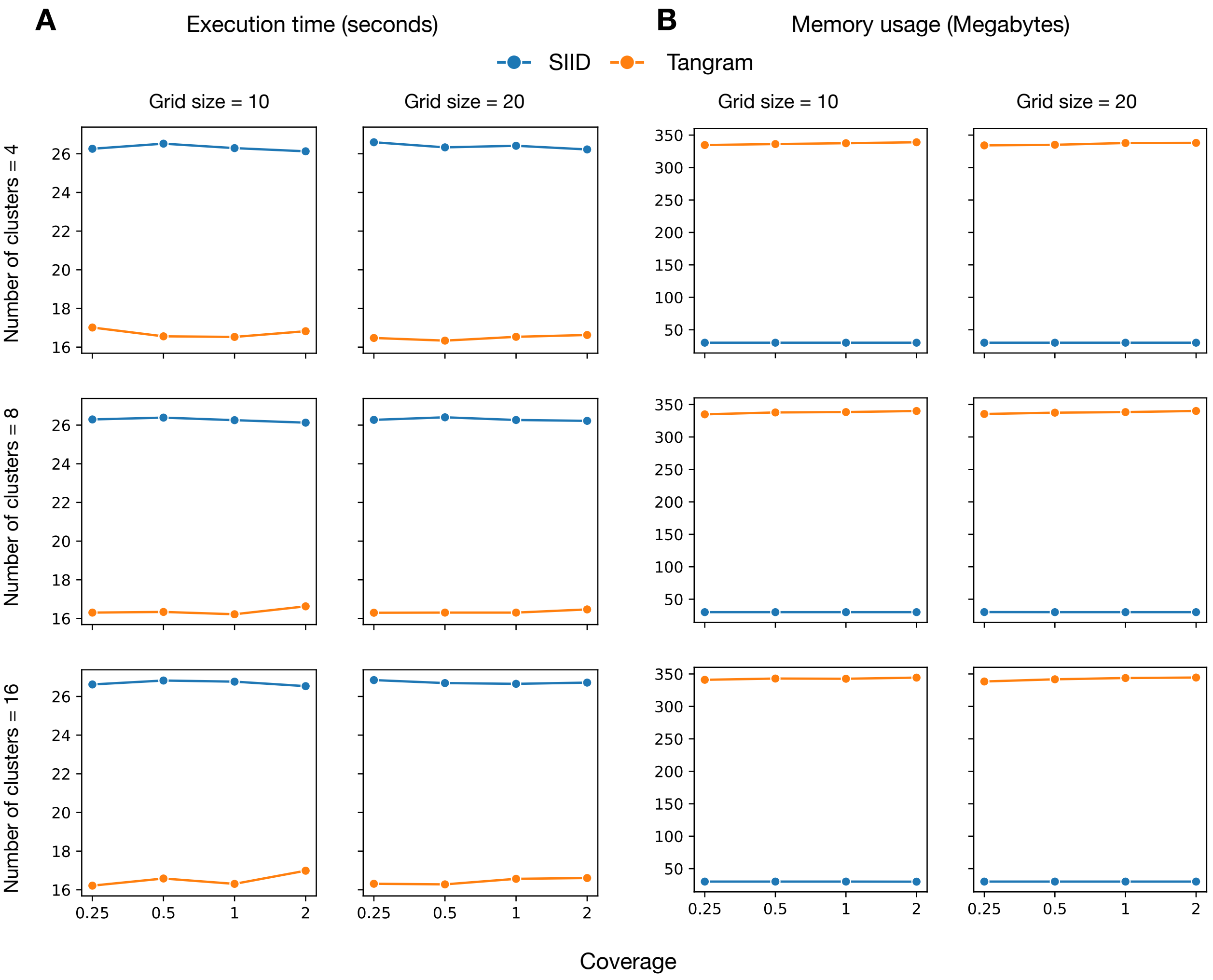

Figure S7: Computational performance comparison of SIID and Tangram across different simulation configurations. **(A)** Execution time (in seconds) for SIID (blue) and Tangram (orange) across varying numbers of clusters (4,8,16), grid sizes (10, 20), and coverage levels (0.25, 0.5, 1, 2). **(B)** Memory usage in megabytes for SIID (blue) and Tangram (orange) under the same simulation setting.

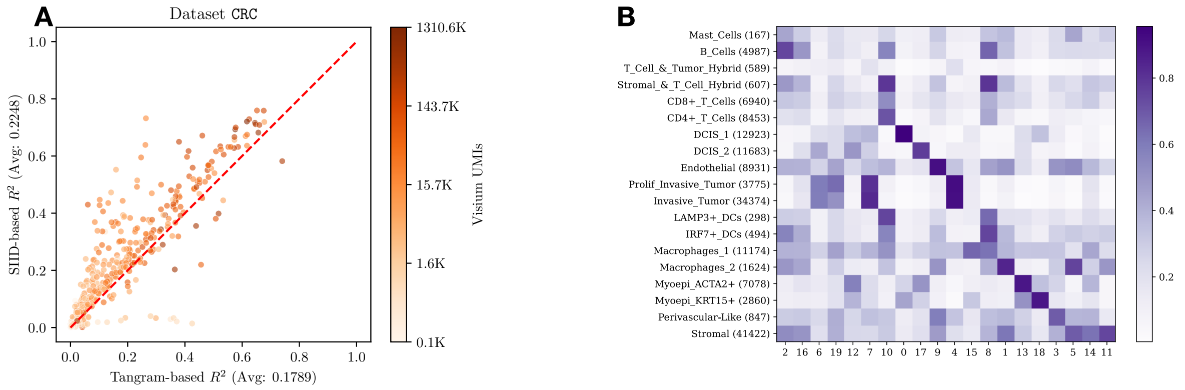

Figure S8: **(A)** Per-gene R^2 score plot for CRC dataset. **(B)** For Visium deconvolution, cosine similarity between inferred cluster proportions from our method (columns) and RCTD inferred cell type proportions (rows). Note that unlabeled cells are discarded when running RCTD.

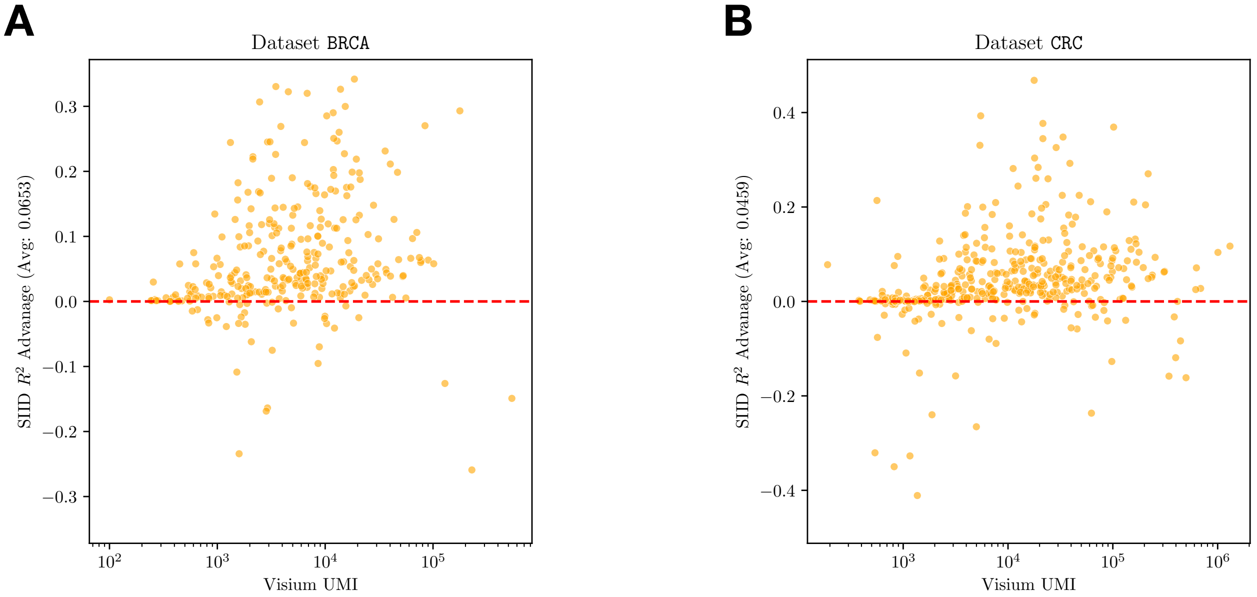

Figure S9: R^2 score differential for each individual gene in both **(A)** BRCA dataset and **(B)** CRC dataset. X-axis represents total UMI counts for each gene in Visium plotted on a log scale. Red dotted line represents $y = 0$ meaning equal R^2 score, dots above the line denote SIID achieving higher R^2 compared to Tangram for that gene.

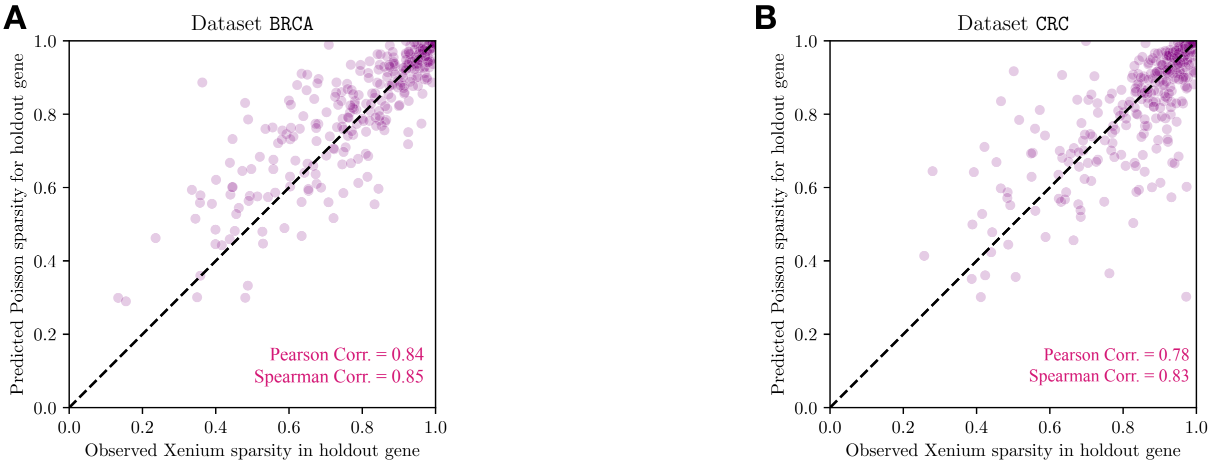

Figure S10: SIID recovers sparsity of Xenium gene expression in **(A)** BRCA dataset and **(B)** CRC dataset. X-axis represents observed sparsity of each gene in the Xenium dataset. Y-axis represents predicted sparsity of each gene in the holdout experiment given $P(Y = 0 \mid Y \sim \text{Pois}(X)) = e^{-X}$ for each observation. Bottom right indicates Pearson and Spearman correlation of sparsity prediction.

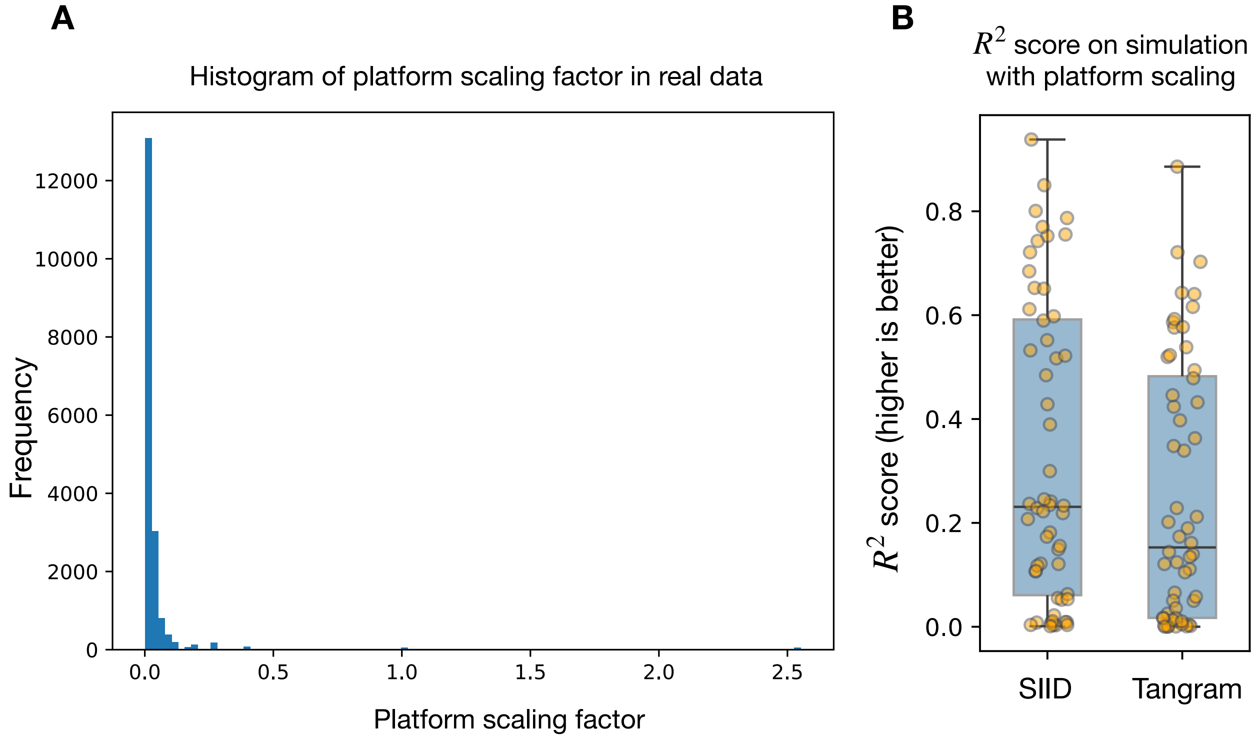

Figure S11: **(A)** Histogram of platform scaling factor for shared genes from BRCA. **(B)** The R^2 score for imputed expression on holdout genes in simulated data with platform specific scaling from SIID and Tangram .

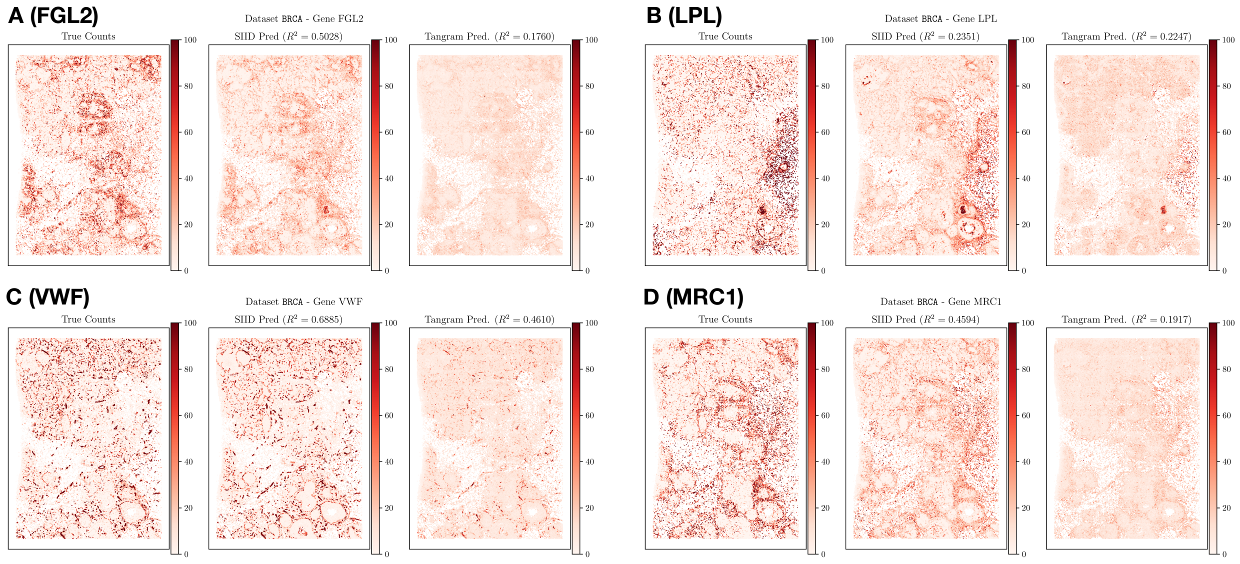

Figure S12: Left: Observed Xenium expression (ground truth for evaluating imputation) Middle: SIID prediction when the gene is held out, Right: Tangram prediction for **(A)** FGL2, **(B)** LPL, **(C)** VWF, and **(D)** MRC1 genes when they are held out. We also present the R^2 scores between predicted expression and observed expression in the title. We normalize the total counts within each gene to $1,000,000$ and plot all normalized counts on the same color scale.

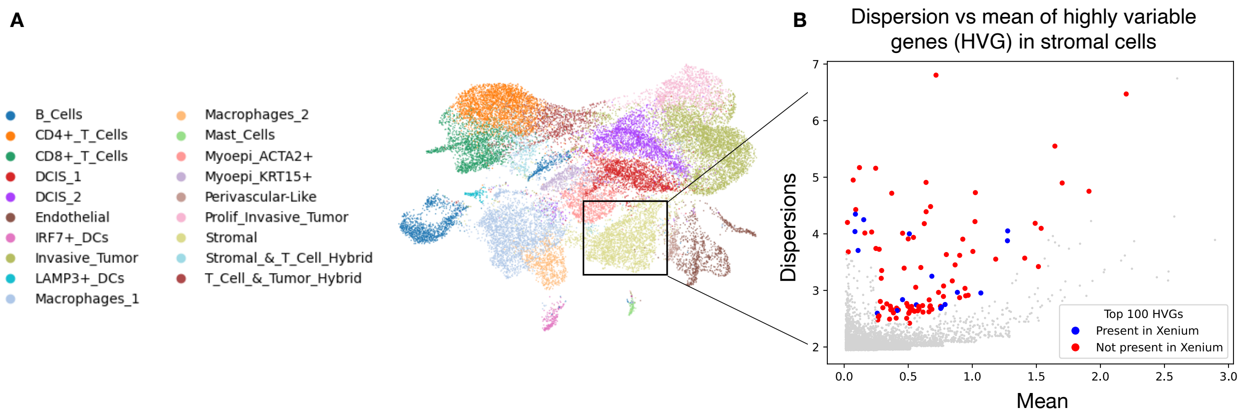

Figure S13: **(A)** A UMAP visualization of annotated cell types in the BRCA scRNA-seq dataset, with box indicating stromal cell population. **(B)** Mean and dispersion of the genes in scRNA-seq data with the 100 most variable genes for the stromal cell type highlighted in blue (present in Xenium panel) or red (not present in Xenium panel).

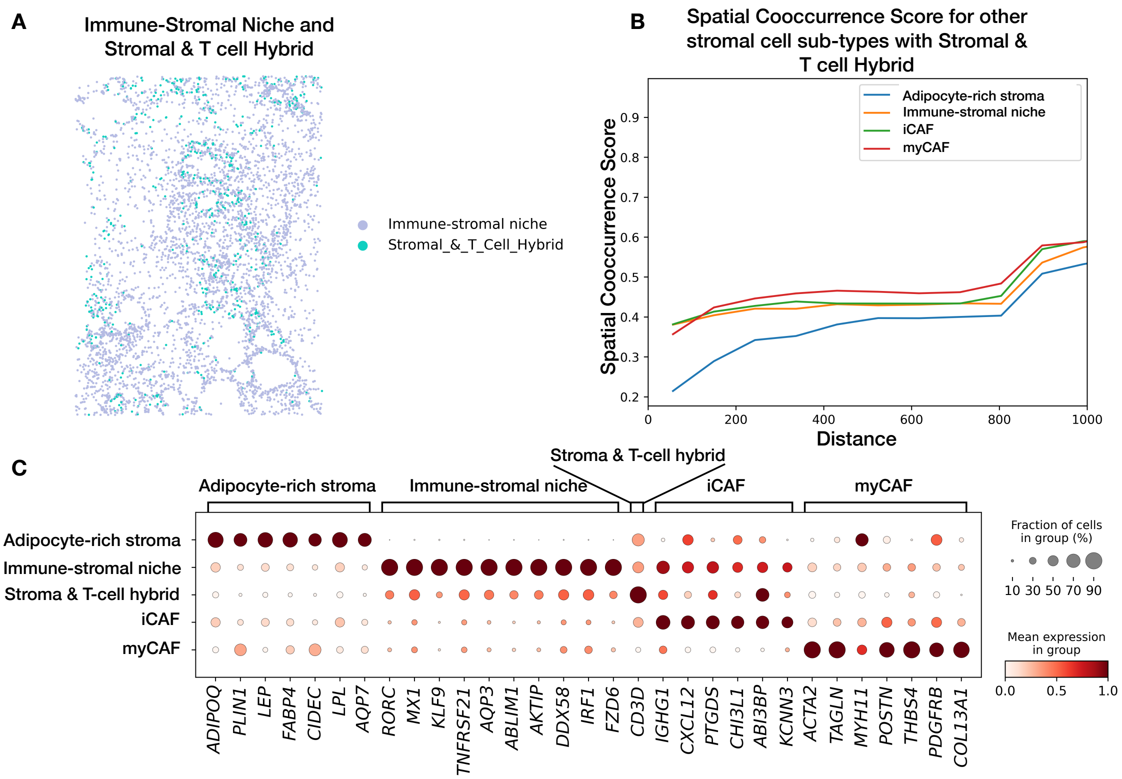

Figure S14: **(A)** Spatial co-localization of Immune-Stromal niche with Stromal and T cell hybrid in BRCA Xenium data. **(B)** Spatial co-occurrence score of stromal cell sub-types with Stromal and T-cell hybrid. **(C)** Marker gene expression for annotated cell subtypes with Stromal and T-cell hybrid.

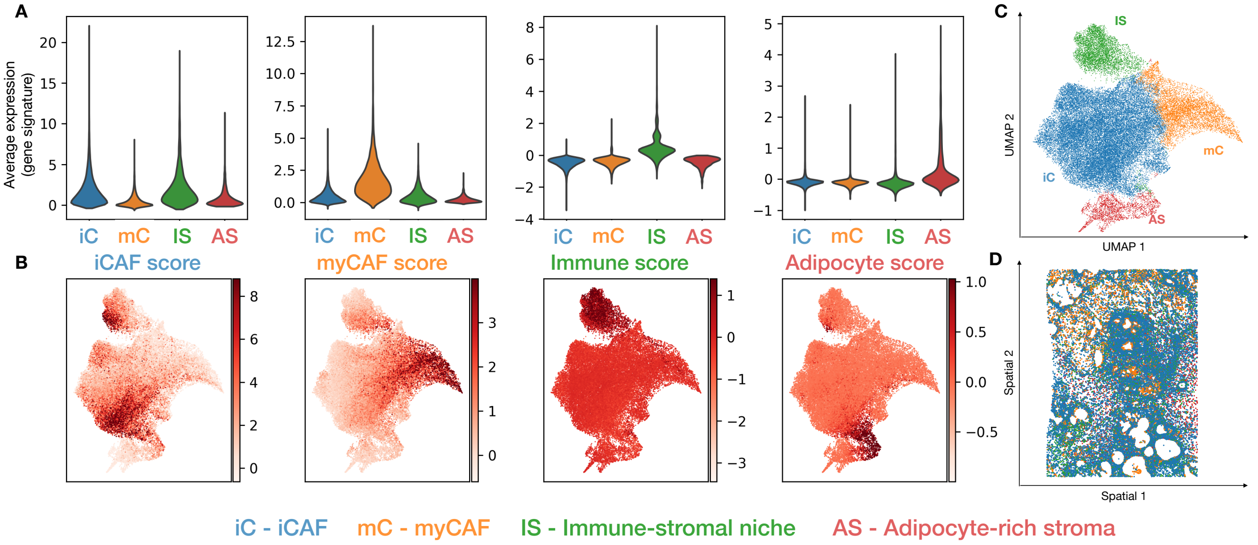

Figure S15: **(A)** Gene set scores for iCAF, myCAF, Immune, and Adipocyte in each of stromal cell subtypes. **(B)** The average expression of genes from the gene sets in each of the corresponding cell subtypes. **(C)** A UMAP embedding of the annotated stromal cell subtypes from Xenium after imputation by SIID BRCA Xenium data. **(D)** Spatial distribution of the four stromal cell subtypes from SIID imputed Xenium data.

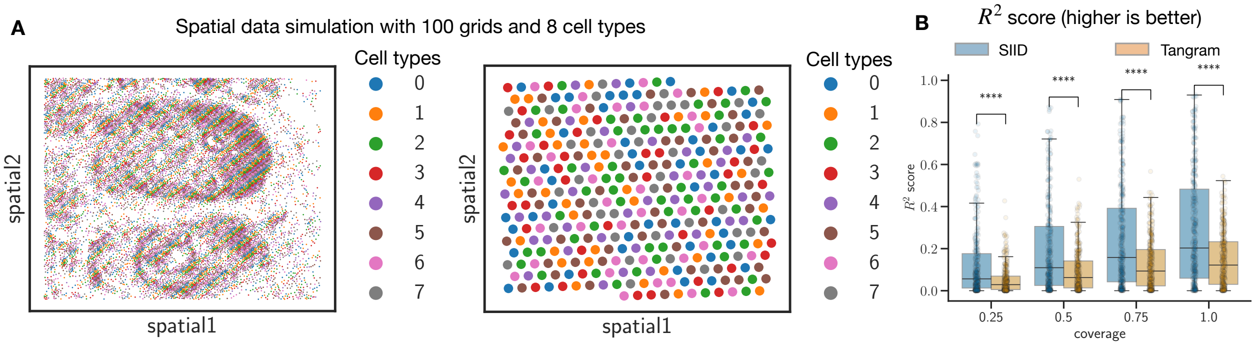

Figure S16: Simulation using $l = 100$ with $h = 8$ cell types. **(A)** Simulated Xenium and Visium data where each location is colored with the cell type color. **(B)** Comparison boxplots of R^2 scores between SIID (blue) and Tangram (brown) on hold out genes at all coverage levels. Statistical significance is indicated by asterisks where **** denotes p-value of $p < 0.0001$.
